# Exploring antibiotic resistance genes and metal resistance genes in plasmid metagenomes from wastewater treatment plants

**DOI:** 10.3389/fmicb.2015.01025

**Published:** 2015-09-24

**Authors:** An-Dong Li, Li-Guan Li, Tong Zhang

**Affiliations:** Environmental Biotechnology Laboratory, Department of Civil Engineering, The University of Hong KongHong Kong, Hong Kong

**Keywords:** plasmid, wastewater treatment, antibiotic resistance, metal resistance, metagenome

## Abstract

Plasmids operate as independent genetic elements in microorganism communities. Through horizontal gene transfer (HGT), they can provide their host microorganisms with important functions such as antibiotic resistance and heavy metal resistance. In this study, six metagenomic libraries were constructed with plasmid DNA extracted from influent, activated sludge (AS) and digested sludge (DS) of two wastewater treatment plants (WWTPs). Compared with the metagenomes of the total DNA extracted from the same sectors of the wastewater treatment plant, the plasmid metagenomes had significantly higher annotation rates, indicating that the functional genes on plasmids are commonly shared by those studied microorganisms. Meanwhile, the plasmid metagenomes also encoded many more genes related to defense mechanisms, including ARGs. Searching against an antibiotic resistance genes (ARGs) database and a metal resistance genes (MRGs) database revealed a broad-spectrum of antibiotic (323 out of a total 618 subtypes) and MRGs (23 out of a total 23 types) on these plasmid metagenomes. The influent plasmid metagenomes contained many more resistance genes (both ARGs and MRGs) than the AS and the DS metagenomes. Sixteen novel plasmids with a complete circular structure that carried these resistance genes were assembled from the plasmid metagenomes. The results of this study demonstrated that the plasmids in WWTPs could be important reservoirs for resistance genes, and may play a significant role in the horizontal transfer of these genes.

## Introduction

Transposable elements can often be accumulated on plasmids that usually act as extra-chromosomal hereditary elements that are physically separated from the chromosomal DNA, and they can replicate themselves independently in cells (Lederberg, 1952; Thomas and Nielsen, 2005). To a large extent, the transformation and conjugation of plasmids play significant roles in the reshuffling of genetic material and horizontal transfer (Koonin and Wolf, 2008). Very early pure culture analyses already indicated that vast prokaryotes can rapidly adapt to their ecological niches through interspecies gene distribution caused by plasmid transfer, including antibiotic resistance (Jacob and Hobbs, 1974), virulence factors (Censini et al., 1996), nitrogen fixation (Bánfalvi et al., 1981), and degradation of toxic compounds (Haugland et al., 1990). These interspecies genes often contribute to the phenotypic diversity of the host and even confer advantages for host survival in their ecological niche (Arraj and Marinus, 1983). However, the mechanistic understanding of the plasmid horizontal gene transfer (HGT) processes is still limited to the analysis of several model organisms (Thomas and Nielsen, 2005) because the majority of microorganisms are not culturable in the laboratory (Amann et al., 1995; Hugenholtz, 2002). Luckily, several studies have been successful in characterizing the genetic diversity of plasmid metagenomes, and have significantly increased our knowledge of their functions by using culture-independent metagenomic approaches (Zhang et al., 2011; Kav et al., 2012; Sentchilo et al., 2013).

Wastewater treatment systems are habitats with a high exchange rate of chemical materials and biological components (Szczepanowski et al., 2008). Taking activated sludge (AS) as an example, it is one of the hotspots for HGT because of its high bacterial diversity (over 700 genera and 3000 OTUs (operational taxonomy unit) at a 97% similarity cutoff) (Zhang et al., 2012) and high density (generally 2–10 g dry weight/L) (Grady et al., 2011) of microorganisms. Meanwhile, wastewater treatment plants (WWTPs) also have been considered as important reservoirs for different types of antibiotic resistance genes (ARGs), which are associated with human pathogens (Rizzo et al., 2013), mainly because municipal WWTPs receive human fecal waste containing various types of antibiotic resistant bacteria. On the other hand, the dense and diverse microbial communities in AS, digested sludge (DS) and influent may accelerate the HGT process of resistance genes including ARGs, metal resistance genes (MRGs) or other important functional genes among different microbial populations. Therefore, microbial communities in WWTP exist as model systems to explore various plasmid carrying genes with important functions for their hosts, such as antibiotic resistance, heavy metal resistance (Van Limbergen et al., 1998; Dröge et al., 2000), biodegradation (Ma et al., 2012), and virulence factors (Mnif et al., 2013). In recent years, various plasmids containing such genes were isolated from microorganisms in WWTPs and analyzed at the genomic level (Szczepanowski et al., 2004; Schlüter et al., 2007). These studies showed that the microorganism communities from WWTPs contained immense varieties of plasmids.

Traditionally, plasmids were sequenced by primer walking and other PCR based methods, which are time-consuming and costly (Alvarez et al., 2004; Jacoby et al., 2006). Meanwhile, researchers also demonstrated the obvious limitations of current plasmid genomics and functional studies, including (1) many plasmids were not replicable in laboratory hosts, (2) the plasmids without selectable marker genes were difficult to capture, and (3) traditional sequencing methods are limited in obtaining the complete circular structure of plasmids in a high-throughput way (Jones, 2013). Compared with traditional methods, the high-throughput sequencing provides much more data in a more rapid way at a lower cost, and makes it feasible to survey the plasmid metagenomes with culture independent approaches (Sentchilo et al., 2013).

**Figure 1:**
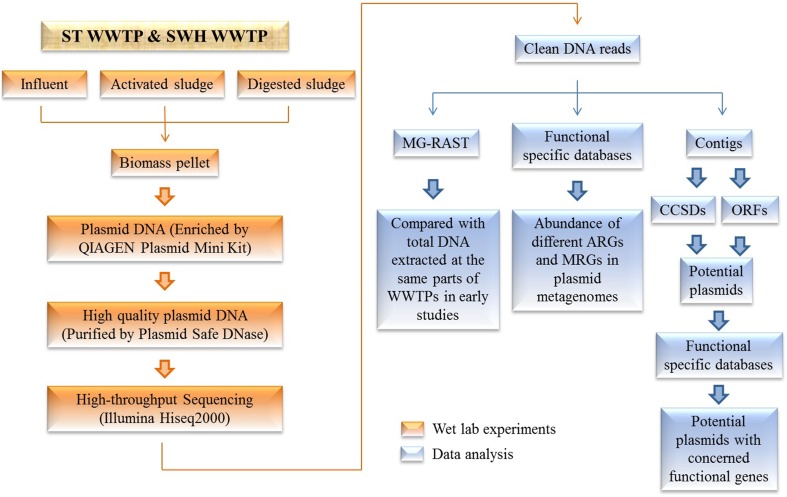
**Schematic of the study workflow**. Plasmid metagenomes were extracted from different sectors of WWTPs followed by purification with Plasmid Safe DNase digestion, as were the subsequent bioinformatics approaches that were used to analyze the plasmid metagenomes.

Here, we report six plasmid metagenomes in the microbial communities from two municipal WWTPs (Figure 1) at great sequencing depth. Four of the corresponding total DNA metagenomes from previous studies (Yang et al., 2013, 2014b,c) were compared with these plasmid metagenomes. Meanwhile, the abundances of ARGs and MRGs in the plasmid metagenomes were analyzed by searching against the antibiotic resistance genes database (ARDB) and the metal resistance genes database (MRDB). Potential plasmids with a complete circular structure were assembled from the plasmid metagenomes (Jørgensen et al., 2014) to characterize the present ARGs and MRGs.

## Materials and methods

### Sample collection

Six samples were collected in March 2013 from different sectors (influent, AS and DS) of two WWTPs of Hong Kong; Shatin Wastewater Treatment Plant (ST WWTP) which treats 230,000 m^3^ sewage and Shek Wu Hui Wastewater Treatment Plant (SWH WWTP) which treats 93,000 m^3^ sewage per day. These plants mainly include domestic sewage but a significant portion of wastewater comes from a slaughterhouse in the catchment area. The samples, designated as SWHAS, SWHDS, SWHIN, STAS, STDS, and STIN (Table S1), were transported to the laboratory within 1 h and were used immediately for plasmid DNA extraction.

### Sludge biomass purification

Biomass in different samples [1 L for AS, 0.2 L for DS and 10 L for influent (based on the biomass concentration in the samples)] was collected by centrifuging at 1000 g (2400 rpm, radius: 155 mm) for 3 min (Sentchilo et al., 2013). The supernatant was decanted and the biomass pellet was re-suspended in ice-cold poly (beta-amino) esters (PBAE) buffer (PBAE buffer is 10 mM Na-phosphate, 10 mM ascorbate, 5 mM EDTA, pH 7.0). Each portion of PBAE-biomass was homogenized by magnetic stirring for 15 min. After that, coarse particles, large bacterial flocks and large eukaryotic cells were removed by low speed centrifugation for 6 min in 50-ml conical tubes at 160 g. The resulting supernatants were centrifuged at 6000 g for 6 min to collect the microbial biomass in the supernatants.

### Plasmid metagenome extraction

The plasmid DNA was extracted by alkali lysis using the QIAGEN Plasmid Mini Kit (QIAGEN Science Inc., Germantown, MD, USA). After precipitating the protein and most of the chromosomal DNA in the system, the extracted plasmid DNA was purified by column binding and washed following the manufacturer's protocol. Finally, the plasmid DNA was harvested by precipitation with isopropanol and washed using 70% ethanol. To further remove the remaining sheared genomic DNA, ATP-dependent Plasmid Safe DNase (Epicentre, USA) was used to treat the extracted plasmid DNA according to the manufacturer's instructions. After overnight incubation at 37°C, the digestion system was inactivated by heating at 70°C for 30 min and subsequently chilled on ice. The plasmid DNA was further precipitated with 3 M sodium acetate (pH 7.0) and washed with 70% ethanol for purification. After drying the plasmid DNA, each plasmid DNA sample was dissolved in 100 μl water. The concentration of DNA in each sample was measured by Qubit 2.0 (Invitrogen Life Technologies).

### High-throughput sequencing

Approximately 5 μg of DNA from each extracted plasmid DNA sample was used for library construction (170 bp insert). High-throughput sequencing was performed at the Beijing Genomics Institute (BGI) with the Illumina Hiseq 2000 platform using the sequencing strategy of the index PE101+8+101 cycle (Paired-End sequencing, 101-bp reads and 8-bp index sequence). The quality of the sequences was checked by Fastqc (http://www.bioinformatics.babraham.ac.uk/projects/fastqc/) and trimmed with the following parameters: (1) removing reads with 3 or more bases of N; (2) removing reads contaminated by 15 or more bases from adapter; (3) removing duplication; (4) removing reads contains consecutive low quality bases (36 bp low than Q20). Approximately 3 Gb (gigabase pairs) of metagenomic data were generated for each of the six plasmid DNA metagenomes, resulting in a total of 18 Gb data.

### Bioinformatic analysis

The portion of types or subtypes of sequences with different functions in “total metagenome sequences” were defined as “abundance” (using the unit of “ppm,” one read in one million reads); (Yang et al., 2013).

#### MG-RAST analysis

Six sets of quality-trimmed reads, i.e., influent from SWH (3.2 Gb) and ST (3.5 Gb), DS from SWH (3.1 Gb) and ST (3.0 Gb), and AS from SWH (2.7 Gb) and ST (2.5 Gb), were uploaded to MG-RAST (Meyer et al., 2008) and annotated with the COG and KEGG databases. Another four sets of quality-trimmed reads [extracted from related total DNA using the FastDNA SPIN Kit for soil (MP Biomedicals, Santa Ana, CA) and sequenced by Illumina Hiseq2000 in early research (DS from SWH: 1.0 Gb; AS from ST: 2.6 Gb; DS from ST: 1.0 Gb, Influent from ST: 1.7 Gb)] were compared together with the plasmid reads by using the Statistical Analysis of Metagenomic Profiles (STAMP) with the *G*-test (W/Yates') and Fisher's test at a *p*-value cutoff of 0.05 (Parks and Beiko, 2010). The MG-RAST COG annotation results of the reads were also analyzed by Principal Coordinates Analysis (PCoA) using Paleonto-logical Statistics Software (PAST); (Hammer et al., 2001).

#### ARGs and MRGs abundance

The plasmid metagenomic datasets were searched against ARDB (Liu and Pop, 2009; Yang et al., 2013) using Usearch with a cutoff of *E*-value: 1e-5 and accel: 0.5 (Yang et al., 2014a). The eligible reads were extracted with customized Perl scripts and searched against the functional specific database again using BLASTX to confirm the annotation accuracy (Yang et al., 2014a). A sequence was annotated as ARGs if its best hit in the related database had ≥90% amino acid identity and the alignment length ≥25 amino acids (Yang et al., 2014a). MRGs annotation was conducted similarly by searching against MRDB (Cai et al., 2013; Li et al., 2014; Pal et al., 2014). The functional sequences were sorted into different types and subtypes of ARGs and MRGs automatically using self-written Python scripts together with the constructed functional specific databases.

### Assembly and gene identification

The plasmid metagenomic reads were assembled using a *de novo* assembly algorithm integrated with three different types of software (Metavelvet 1.2.01, SOAPdenovo and CLC bio Genomic Workbench 6.0.2). CLC bio Genomics Workbench version 6.0.2 (CLC Bio, Aarhus, Denmark) performed the best. Different k-mer sets were tested and k-mer 23 was finally chosen for the assembly. Contigs larger than 500 bp [considering the minimum plasmid length was 471 bp in the NCBI plasmid refseq database (version: 25/7/2013)] were picked for further analysis.

Contigs with the same DNA sequence at the beginning and end were selected using a self-written Python script (available at the website: http://web.hku.hk/~zhangt/ZhangT.htm) and were considered as the sequences of “potential closed circular supercoiled DNAs” (CCSDs) (Bengtsson-Palme et al., 2014). MetaGeneMark was used to predict the open reading frames (ORFs) located on all of the contigs including the CCSDs (Zhu et al., 2010). To analyze the potential function of the ORFs, amino acid sequences of the ORFs located on the CCSDs were used to search against the National Center for Biotechnology Information protein Non-redundant (NCBI-nr) database using BLASTP with default parameters and an *e*-value cutoff of 0.01 (Feil et al., 2005).

For the evaluation of the CCSD assembly efficiency, 22 plasmids smaller than 150 kb (Table S2) were randomly selected from the NCBI refseq plasmid database as reference plasmids and ART linux64 Illumina_src-1.5.1 (Huang et al., 2012) was used to make the plasmid sequences into illumine short reads with different coverage. Because plasmids larger than 150 kb will be poorly extracted using the above extraction method, large plasmid contigs in the plasmid database were not selected as reference plasmids.

CCSDs carrying plasmid maintenance functional related genes were picked out as “potential plasmids” by manually checking based on the description of the BLAST hits. Further analysis of these potential plasmids was performed with BLASTX against the functional specific databases (ARDB and MRDB) with default parameters and an *e*-value cutoff of 0.01. The ARG-like and MRG-like ORFs located on the potential plasmid were manually double-checked for more accurate annotation according to the results obtained by BLASTP against the NCBI-nr database and the specific databases (i.e., ARGD and MRDB). To calculate the average coverage of the plasmids in the plasmid metagenomes, sequenced reads in the six datasets were mapped to the obtained potential plasmid contigs with strict criteria (i.e., 98% similarity and 80% alignment length).

### Accession number

All of the original data sets were uploaded to MG-RAST and described in Table S1 with the following accession numbers: 4537905.3 (STAS_P); 4537907.3; STIN_P: 4537909.3 (STDS_P); 4537911.3 (SWHAS_P); 4537913.3 (SWHDS_P); 4537915.3 (SWHIN_P).

## Results

### Metagenomic analysis of plasmid DNA

Approximately 3 Gb of metagenomic sequences was generated for each plasmid DNA sample, resulting in a total of 18 Gb of data with 180 million clean reads (Table 1). To understand the difference between the plasmid DNA and the total DNA in the same type of samples, four metagenomic datasets of total DNA extracted from AS, DS, and influent of ST WWTP and DS of SWH WWTP were selected from our previous works (Yang et al., 2013, 2014b) to analyze together with the related plasmid metagenomes by searching against the KEGG database on MG-RAST. Not surprisingly, amino acid metabolism, carbohydrate metabolism and genetic information processing were the most dominant types of functions.

**Table 1:** **Numbers and abundance of ARGs-like and MRGs-like reads in the six plasmid metagenomes**.

**Samples**	**Clean reads**	**Contigs (>500 bp)**	**ARG-like reads**	**Abundance of ARGs (ppm)**	**MRG-like reads**	**Abundance of MRGs (ppm)**
STAS_P	24,597,746	46,965	1693	69	1330	54
STDS_P	30,037,254	46,995	1312	44	391	13
STIN_P	35,018,772	64,930	24,794	708	6437	184
SWHAS_P	27,385,286	44,397	2414	88	1411	52
SWHDS_P	31,211,060	58,917	2464	79	1122	36
SWHIN_P	31,892,532	66,454	16,312	511	5744	180

**Figure 2:**
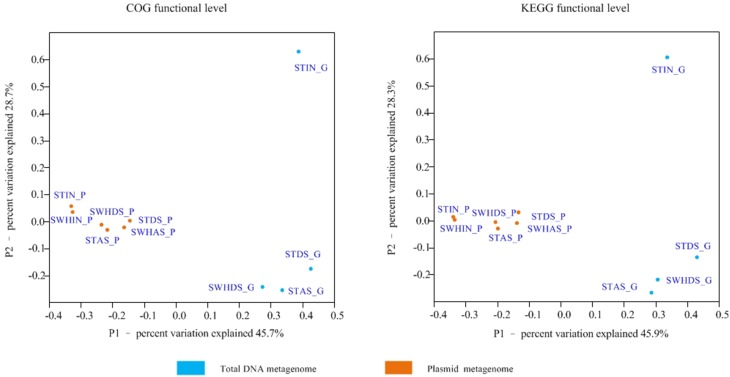
**PCoA analysis at the read level with the Bray-Cutis algorithm (based on the MG-RAST COG & KEGG functional level annotation)**.

**Figure 3:**
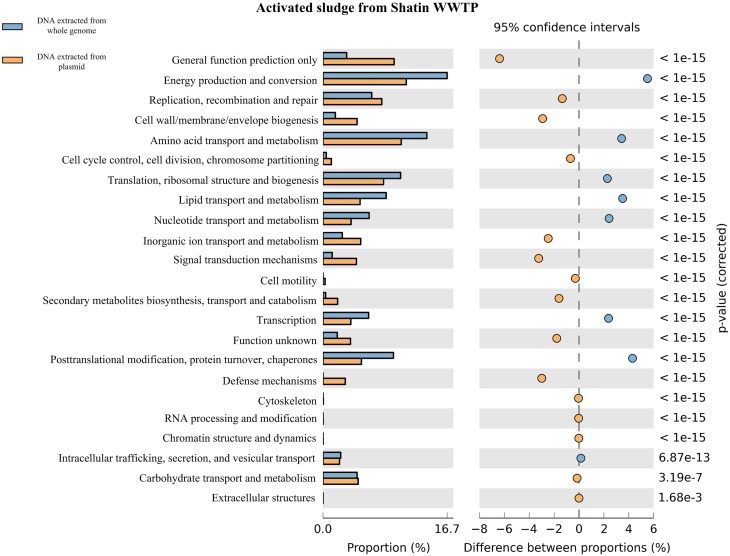
**Functional genes in the plasmid metagenome vs. the genes in the total DNA metagenome extracted from STAS based on the percentages of COG categorized genes and pairwise proportional differences calculated using STAMP (Statistical Analysis of Metagenomic Profiles)**.

In addition to the comparison based on KEGG annotation, a further comparison of the annotated genes of the plasmid metagenome and those of total DNA was conducted using PCoA analysis based on the COGs annotation with the Bray-Cutis algorithm (Figure 2). It showed the unique dispersion of plasmid metagenomes. The six plasmid metagenomes clustered together, while the metagenomes of total DNA were scattered, especially the Influent metagenome. Recent observations demonstrated that some special types of proteins such as secreted proteins were adaptive and cooperated with prokaryotic traits selected on mobile genetic elements (Rankin et al., 2011). Furthermore, plasmid metagenomes had a higher density of mobile genetic elements than the total DNA metagenomes (Zhang et al., 2011). Meanwhile, the comparison of the functional genes in the plasmid metagenomes and the total DNA metagenomes were shown in Figure 3.

**Figure 4:**
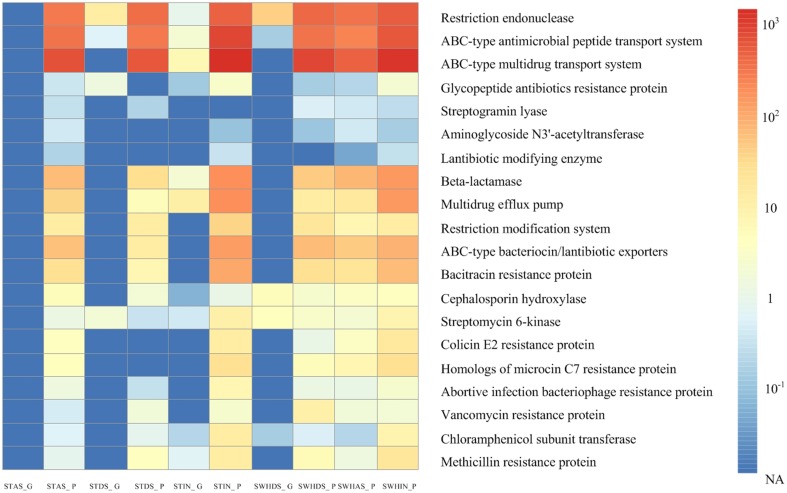
**Comparison of the plasmid metagenome to the total DNA metagenome based on the abundance of the defense mechanisms obtained using the COG database of MG-RAST**. Every lattice represents the abundance of the reads.

To explore the roles of plasmids in their hosts, we chose the genes related to defense mechanisms for further analysis. As displayed in Figure 4, it was obvious that, compared with the total DNA metagenomes, the plasmid metagenomes were significantly rich in genes related to defense mechanisms. As expected, the genes about the restriction endonuclease and modification system were enriched in the plasmid metagenomes. On the other hand, the defense mechanisms described herein are mostly related to antibiotic resistance among the different detected ARG types; the ABC transport system and the cation efflux pump genes which usually work as multidrug resistance genes were the most dominant genes in all of the plasmid metagenomes.

### Occurrence and abundance of different types of ARGs and MRGs

Through the application of metagenomic approaches, a more comprehensive profile of the types and subtypes of ARGs and MRGs can be investigated as they are listed in the functional specific database. Therefore, types together with subtypes for ARGs or MRGs in different metagenomes can be compared together in a united platform without amplification bias. In the six plasmid metagenomes, we found a total of 48,989 reads matching the ARGs and 16,435 reads matching the MRGs.

Table 1 listed the abundances of ARG-like and MRG-like sequences in each plasmid metagenome. The ARGs ranged from 44 ppm (STDS_P) to 708 ppm (STIN_P), while the MRGs ranged from 13 ppm (STDS_P) to 184 ppm (STIN_P). Generally, the abundances of ARGs and MRGs in the influent plasmid metagenomes were much higher than those in the other metagenomes, and the DS plasmid metagenomes had the lowest ARG and MRG abundances. These were consistent with the MG-RAST analysis and revealed the fates of these genes in WWTPs (Yang et al., 2014b).

#### Antibiotic resistance genes

**Figure 5:**
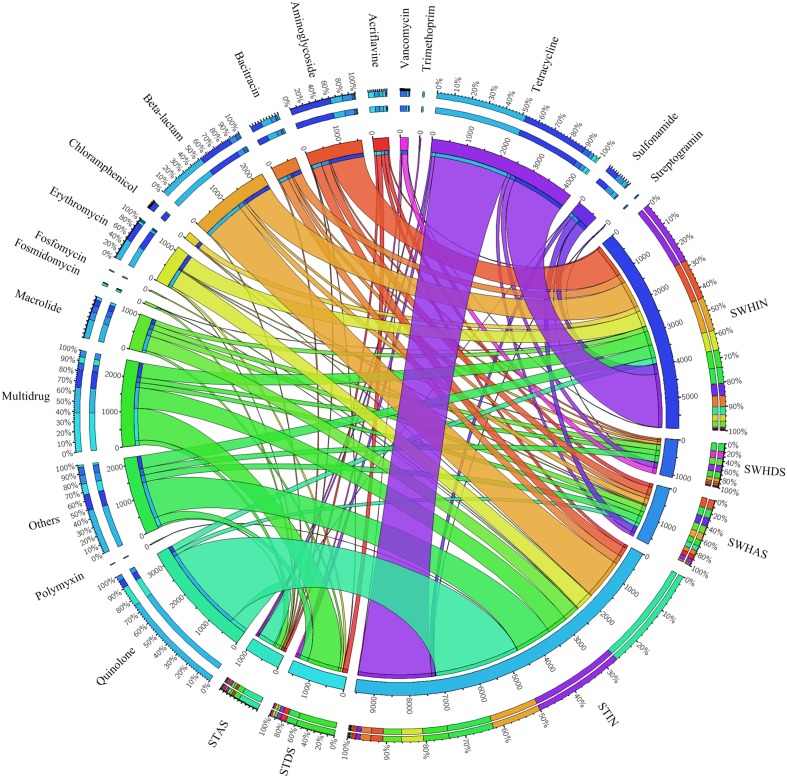
**Distributions of ARG types and their abundances in the total annotated ARGs in the six plasmid metagenomes (visualized ***via*** Circos)**. The length of the bars on the outer-ring represents the percentage of ARGs in each plasmid metagenome. Each ARG was represented by a specific ribbon color, and the width of each ribbon demonstrates the abundance of each ARG in the plasmid samples.

Figure 5 showed the dispersion of the different types of ARG-like sequences in the six plasmid metagenomes. A total of 18 types of ARGs were detected in all of the plasmid metagenomes among the 25 types in the structured ARDB database. Among all of the detected ARGs, the most abundant type was tetracycline resistance genes (29%), followed by quinolone resistance gene (17%), beta-lactam resistance genes (12%), aminoglycoside resistance genes (10%) and the resistance genes of macrolide-lincosamide-streptogramin (10%).

#### Occurrence and abundance of ARGs subtypes

A more comprehensive profile of ARGs subtypes in plasmid metagenomes showed that a total of 323 ARGs subtypes were identified and the top 10 ARGs subtypes accounted for more than 45% of the identified ARGs sequences. Among them, there were four subtypes of tetracycline resistance genes, two quinolone resistance genes, one macrolide resistance gene, one aminoglycoside resistance gene, one sulfonamide resistance gene, and one ARG annotated as a hypothetical protein (Tables S3, S4). The “quinolone resistance protein” contributed the most in the subtype of ARGs (14%) and the “ribosomal protection tetracycline resistance protein” contributed the second most (9%). On the other hand, in the four total DNA metagenomes, a total of 362 ARGs subtypes were identified and the top 10 ARGs subtypes accounted for approximately 40% of the identified ARGs sequences. In addition to the “hypothetical protein” (9%), the “tetracycline resistance protein” contributed the most (6%).

#### Heavy metal resistance genes

Figure S4 displayed the abundances of the MRG-like sequences in the plasmid metagenomes. All types of heavy MRGs listed in the MRDB were detected in the plasmid metagenomes. The zinc and copper resistance genes were the two most dominant (22 and 20%, respectively) types in all of the detected MRG-like sequences, followed by resistance genes for cobalt (11%) and arsenic (11%).

### Plasmid identification

The assembly test results using three types of assembly software (Metavelvet 1.2.01, SOAPdenovo and CLC bio Genomic Workbench 6.0.2) with different K-mer sets were shown in Tables S5–S7. CLC bio Genomic Workbench 6.0.2 with 23 K-mer sets performed best. A total of 328,658 contigs (>500 bp) were assembled by using the CLC bio Genomics Workbench with the 23 K-mer set (Table 1). From the above contigs, 404 contigs were identified as having a closed circular structure and defined as CCSDs. Based on the ORF annotation results, 114 CCSDs contained functional plasmid genes and were considered as potential plasmids (Table S8). Among them, five potential plasmids carrying ARGs and 12 plasmids carrying MRGs were picked out based on the BLASTX results against their specific databases, including one plasmid carrying both ARG and MRG. As shown in Figures S5–S10, the sizes of five ARGs-containing plasmids were from 2 to 5 kb and there were few genes encoding functions other than antibiotic resistance or plasmid maintenance functions. Meanwhile, the sizes of potential plasmids which would encode MRGs were from 1 to 11 kb (Figures S11–S23).

## Discussion

In this work, we successfully used metagenomic data to describe plasmid metagenomes extracted from influent, AS and DS from two municipal WWTPs. By comparing with the total DNA metagenomes extracted in our previous works (Yang et al., 2013, 2014b), the plasmid metagenomes were significantly distinguished from the total DNA metagenomes (Figure 2). Based on the MG-RAST analysis, the annotation rates of the reads of plasmid metagenomes were much higher than those of the total DNA metagenomes (Table S9). This may be because the functional genes on plasmids are commonly shared by those cultured microorganisms and were already better characterized in the KEGG database. Meanwhile, genes for defense mechanisms, including mainly ARGs, were significantly enriched in the plasmid metagenomes (Figure 4), indicating that the ARGs were enriched in the plasmid metagenomes and highlighting the HGT of ARGs encoded on plasmids (Zhang et al., 2011). Meanwhile, the DNA extracted from the influent samples, which contained high levels of human fecal bacteria, contained more genes for defense mechanisms than samples extracted from AS and DS (both plasmid metagenomes and the total DNA metagenomes). This is in agreement with previous studies on the fates of ARGs in the treatment processes of the WWTPs (Yang et al., 2014b).

As shown in Figure 3 and Figures S1–S3, the total DNA metagenomes contained much more basic functional genes related to the metabolism of amino acids or protein, energy production or conversion, etc. Meanwhile, based on the COG category annotation, plasmid metagenomes had more functional genes for signal transport, inorganic ion transport and defense mechanisms, indicating that these types of genes are more easily transferred *via* HGT in the microbial community. On the other hand, plasmid metagenomes contained more unknown functional genes compared with the total DNA metagenome. These results were in agreement with previous studies (Kav et al., 2012; Sentchilo et al., 2013) and demonstrated that plasmid metagenomics is an efficient approach for functional gene study in microbial communities.

### ARGs and MRGs in plasmid metagenomes

From the ARG type and subtype analysis, we found that some ARGs such as those resistant to tetracycline, quinolone and beta-lactam, which are most commonly in our daily lives, had the highest abundance in the plasmid metagenomes. Tetracycline resistance contributed to nearly 30% of the detected ARGs in the influent plasmid samples, which was because tetracycline is widely used in both human medical treatment and the animal breeding industry (Bryan et al., 2004); thus, it is not surprising that we observed the relatively high levels of tetracycline resistance genes in the influent samples. Meanwhile, compared with the total DNA metagenomes, the distribution of ARG subtypes in the plasmid metagenomes was more inclined to be focused on specific subtypes. Specifically, the genes which encoded “quinolone resistance protein” were enriched largely in the plasmid metagenomes, especially the STIN plasmid metagenome. The quinolone resistance gene had been considered to only be acquired through mutation or vertical transmission until the first plasmid encoding a quinolone resistance gene was detected in the late 1990s (Robicsek et al., 2006), indicating that before the 1990s, the abundance of the plasmid carrying quinolone resistance genes might not be as high in abundance as today. Based on the data listed in Table 1, we may conclude that human contact systems, such as sewage influent, contribute largely to the HGT of plasmids carrying ARGs.

In all of the detected MRGs, the resistance genes for zinc, copper, and cobalt were quite widespread in environment because these metal ions are essentially required nutrients for microorganisms, yet at high concentrations they can be toxic (Ji and Silver, 1995). Meanwhile, copper and arsenic are widely used in combination in antibiotics in the animal breeding industry. This could also contribute to the high abundance of copper and arsenic resistance genes in the plasmid metagenomes, especially in SWH WWTP which has slaughterhouse wastewater in its influent.

The abundance of the identified potential plasmids in the six plasmid metagenomes was shown in Table S10. Five plasmids carrying ARGs were selected from the STIN and SWHAS plasmid metagenomes. DS plasmid datasets had a much lower abundance of these five plasmids than the metagenomes extracted form AS and influent samples. Three of them had the highest abundance in the STIN dataset. Meanwhile, the plasmid SWHAS_4082 (Figures S9, S18), which carries both ARG and MRG, was only detected in the AS systems, indicating that antibiotics and metal ions would be accumulated in some specific environmental niches of the AS system such as the different layers of sludge flocs.

Several decades ago, studies using transformation, plasmid capturing and sequencing demonstrated that MRGs and ARGs were linked (Baker-Austin et al., 2006; Seiler and Berendonk, 2012), especially on plasmids (Nakahara et al., 1977), because of antibiotic-metal resistance co-selection. In this study, high-throughput sequencing in conjunction with bioinformatics analysis provided additional evidence for co-resistance against antibiotics and heavy metals. This could be the reason that the distribution pattern for the abundance of MRGs in each plasmid metagenome was similar to ARGs in that the influent metagenomes contained the most MRGs and ARGs while the DS metagenomes contained the fewest.

In sum, a relatively broad range and high abundance of known resistance genes were identified in this study. It would be surprising not to find yet undiscovered resistance genes in such a complex environment. Many ARGs encountered in clinical settings are thought to originate from environmental bacteria through HGT, especially in settings where humans or animals interact (Forsberg et al., 2014). In such situations, there is an obvious risk that ARGs or MRGs selected in the wastewater treatment processes might spread into other water or soil environments, especially those on plasmids. Our data highlighted the significance of the plasmids for the dispersion of ARGs and MRGs in different microbial communities.

### Limitation of the approach

Plasmids carrying ARGs and MRGs have been studied for several decades. However, large plasmids, commonly associated with ARG and virulence genes in clinical and environmental isolates, are difficult to isolate by lab techniques. Taking plasmids isolated from WWTP as an example, Rahube et al. cured four plasmids which were resistant to erythromycin. Compared with the plasmids obtained in their study, with lengths from 20,914 to 87,419 bp (Rahube et al., 2014), we did not obtain large complete plasmids with the ARGs and/or MRGs; the ARGs were particularly difficult.

One of possible reasons is that large plasmids would be heavy burdens for the metabolism of their host and are easy to lose during the reproduction of the microorganisms (Rysz et al., 2013). Therefore, during phylogenetic evolution, small plasmids encoding important auxiliary functions are inclined to be retained. Additionally, antibiotic resistance is usually encoded by single genes (Forsberg et al., 2014). This also leads to the fact that, for most conditions, small sized plasmids will more easily have higher copy numbers than larger plasmids (Sørensen et al., 2005). Thus, during plasmid extraction, smaller plasmids tend to be more enriched than larger plasmids.

However, other biases during the experimental approach may also occur. After collecting the biomass from different samples, we chose the QIAGEN Plasmid Mini Kit (alkaline lysis together with resin purification) to perform plasmid extraction, and the ATP-dependent Plasmid Safe DNase to remove residual chromosomal DNA. This procedure will omit plasmids larger than 150 kb according to the instructions from the manufacturer. What's more, although pre-warming (65°C) elution buffer was used to enhance the elution efficiency, plasmids larger than 45–50 kb would exhibit somewhat reduced elution efficiencies.

Assembly could be another factor of concern in retrieving plasmids from metagenomic data. Different types of software were evaluated in this study and CLC bio Genomics Workbench was finally chosen considering the CCSD acquiring efficiency (Tables S4–S7). For the evaluation the CCSD assembly efficiency, 22 plasmids smaller than 150 kb (Table S2) were randomly selected. The reference plasmid assembly results (Tables S11, S12) agreed with the results of the previous assembly in that the 23 K-mer set showed the best assembly efficiency. These results indicated that read coverage and the arrangement of bases would influence the plasmid assembly results. Furthermore, small size reference plasmids could easily be retrieved as CCSD after assembly. Generally, 12 out of 22 reference plasmids could be retrieved at different coverages with 23 K-mer sets, and all were smaller than 60 kb.

The identification of a potential plasmid was based on the manual checking against the annotation of the ORFs. In addition to the possible mistakes caused by manual errors, bias may also arise from the used database or algorithm. Although the NCBI database updates continually, many proteins in nature remain unknown. This would result in the amount of potential plasmids being underestimated in this study and other studies using a similar approach.

### Contribution and significance

With the pipeline described in this article, we successfully obtained plasmid metagenomes from influent, AS and DS of WWTPs without the biases of culture-based methods. To extract plasmids, traditional plasmid methods were used: alkaline lysis or hot alkali lysis in combination with acid phenol–chloroform treatment followed by isopycnic density centrifugation in a CsCl-ethidium bromide gradient (Sentchilo et al., 2013). In this study, we used alkaline lysis together with resin purification followed by the ATP-dependent Plasmid Safe DNase treatment. The traditional plasmid extraction procedures are very time-consuming. For instance, isopycnic density centrifugation will take 48 h. However, the entire plasmid extraction methods proposed in this study could be finished in 8 h.

By using high-throughput sequencing, we described the plasmid metagenomes of relatively high depth from six different microbial communities and recovered a number of potential plasmids with important resistance genes. The metagenomic analysis also provided a broad-spectrum scan of various types and subtypes of ARGs and MRGs in plasmid metagenomes of three typical microbial communities in WWTPs. Because of widely emerging of pathogens with different resistance genes, more studies will be performed on such genes and their mobility in human contacted areas. The approaches proposed in this study will continually provide novel insights on the fates of plasmids in different microbial communities.

### Conflict of interest statement

The authors declare that the research was conducted in the absence of any commercial or financial relationships that could be construed as a potential conflict of interest.
