# Supplementary material for: Exploring antibiotic resistance genes and metal resistance genes in plasmid metagenomes from wastewater treatment plants
Source: Front Microbiol. 2015 Sep 24;6:1025. doi: 10.3389/fmicb.2015.01025 (PMC4585309; doi:10.3389/fmicb.2015.01025)
Supplement: Supplementary file 1 [file DataSheet1.PDF]

Table S1. Summary of accession codes and sample description

Name	Accession number	Sample description
STAS_P	4537905.3	Plasmid DNA extracted from AS of ST WWTP
STDS_P	4537907.3	Plasmid DNA extracted from DS of ST WWTP
STIN_P	4537909.3	Plasmid DNA extracted from influent of ST WWTP
SWHAS_P	4537911.3	Plasmid DNA extracted from AS of SWH WWTP
SWHDS_P	4537913.3	Plasmid DNA extracted from DS of SWH WWTP
SWHIN_P	4537915.3	Plasmid DNA extracted from influent of SWH WWTP
STAS_G[1]	4489101.3	Total DNA extracted from AS of ST WWTP
STDS_G	4489101.3	Total DNA extracted from AS of ST WWTP
STIN_G[2]	4497219.3<br>4497229.3	Total DNA extracted from influent of ST WWTP
SWHDS_G	4489062.3	Total DNA extracted from DS of SWH WWTP

Table S2. Reference plasmids for assembling evaluation

No.	Seq-ID	length (bp)
PC1	gi|125654605|ref|NC_009007.1|	114045
PC2	gi|217975547|ref|NC_011664.1|	88311
PC3	gi|283826834|ref|NC_013727.1|	70275
PC4	gi|224796457|ref|NC_012164.1|	56055
PC5	gi|296137611|ref|NC_014154.1|	45943
PC6	gi|148244055|ref|NC_009470.1|	37415
PC7	gi|219364539|ref|NC_011790.1|	31266
PC8	gi|218442241|ref|NC_011730.1|	29239
PC9	gi|186686638|ref|NC_010629.1|	26419
PC10	gi|32455473|ref|NC_004966.1|	20331
PC11	gi|172034885|ref|NC_010542.1|	14685
PC12	gi|85060490|ref|NC_007715.1|	10810
PC13	gi|60461906|ref|NC_000906.2|	8506
PC14	gi|284005992|ref|NC_013737.1|	7308
PC15	gi|10956144|ref|NC_001763.1|	6024
PC16	gi|49176958|ref|NC_005910.1|	5113
PC17	gi|222143325|ref|NC_012000.1|	4398
PC18	gi|23307864|ref|NC_004253.1|	3661
PC19	gi|10956578|ref|NC_001456.1|	3054
PC20	gi|190410158|ref|NC_010871.1|	2248
PC21	gi|10957087|ref|NC_001316.1|	1313
PC22	gi|56687495|ref|NC_006528.1|	846

Table S3. Top 10 subtype ARGs in plasmid metagenomes (ppm)

Subtype name	STAS_P	STDS_P	STIN_P	SWHAS_P	SWHDS_P	SWHIN_P
Quinolone resistance protein	26.71	1.03	172.65	3.03	0.26	11.95
Ribosomal protection tetracycline resistance protein	0.69	0.33	25.02	0.29	2.98	100.31
Hypothetical protein	3.46	3.00	32.98	2.67	5.41	18.59
Tetracycline resistance protein	1.59	3.10	20.47	2.67	5.54	20.91
TetQ	1.63	0.20	36.32	0.26	0.35	15.52
ErmB	0.85	0.50	27.59	1.10	1.35	9.75
Tet32	0.69	1.73	14.73	0.58	3.20	13.32
QnrS	4.11	0.20	25.39	0.40	0.03	1.38
Aminoglycoside acetyltransferase	1.10	0.03	5.25	10.01	1.41	12.42
Sul1	1.26	0.47	4.74	7.19	1.15	13.23

Table S4. Top 10 subtype ARGs in total DNA metagenomes (ppm)

Subtype name	STAS_G	STDS_G	STIN_G	SWHDS_G
Hypothetical protein	1.99	5.78	67.56	5.16
Tetracycline resistance protein	1.33	6.92	44.23	3.27
Quinolone resistance protein	0.04	1.76	40.00	0
AcrB	1.83	3.14	35.03	1.38
TetW	0.23	5.66	27.38	2.14
Tet32	0.55	2.01	23.27	2.64
Amphiphile efflux-1 (HAE1) family protein	1.68	3.14	16.93	1.63
TetM	0.31	3.90	16.01	1.38
Multidrug efflux protein	0.98	1.76	16.44	1.38
StrA	0.90	2.01	15.63	1.63

Table S5. Assembly evaluation of different K-mer sets with CLC Genomic Workbench using plasmid metagenome extracted from SWHAS

**K-mer**	**23**	**29**	**35**	**51**	**57**	**63**
N50	1092	1078	1078	920	841	798
No. of CCSD	129	115	105	93	86	80

Table S6. Assembly evaluation of different K-mer sets with Metavelvet using plasmid metagenome extracted from SWHAS

**K-mer**	**23**	**45**	**51**	**57**	**63**
N50	603	686	866	826	775
No. of CCSD	1	23	81	75	72

Table S7. Assembly evaluation of different K-mer sets with SOAPdenovo using plasmid metagenome extracted from SWHAS

**K-mer**	**23**	**31**	**35**	**43**	**49**
N50	722	987	1393	1561	1181
No. of CCSD	11	10	12	9	1

Table S8. Distribution of the potential plasmids and the plasmid functional genes in the plasmid metagenomes

Metagenome	Potential plasmid	Replication related genes	Transposase related genes	Recombination related genes	Integrase related genes	Other plasmid functional genes
STAS	15	11	3	3	1	1
STDS	7	2	3	1	0	2
STIN	20	12	4	4	2	3
SWHAS	45	25	7	5	10	14
SWHDS	17	5	8	1	4	3
SWHIN	10	2	7	1	1	1

Table S9. Annotation summary provided by searching against KEGG database on MG-RAST

Sample Name	Total reads	Annotated reads	Annotation rate
STAS_G	25,636,673	716,084	2.79%
STAS_P	24,597,746	1,814,716	7.38%
STDS_G	7,951,670	135,140	1.70%
STDS_P	30,037,254	1,656,639	5.52%
STIN_G	34,545,912	102,639	0.30%
STIN_P	35,018,772	5,254,537	15.00%
SWHDS_G	7,952,280	240,149	3.02%
SWHDS_P	31,211,060	2,256,804	7.23%

Table S10. Abundances (coverage) of the identified potential plasmids

Name	Contig length	SWHIN	SWHAS	SWHDS	STIN	STAS	STDS
STAS_2085_MRG	11517	0	0	0	0.395	17.539	0.19
STAS_2494_MRG	4019	0	0.15	0	0.717	33.551	0.07
STDS_12284_MRG	1852	0	0	0	0.054	0	15.49
SWHAS_2999_MRG	8099	11.08	81.93	0.31	0.208	0.367	0.03
SWHAS_3486_MRG	1866	1.13	17.37	0.27	0	0	0
SWHAS_4504_MRG	1624	0.91	27.95	0	0	0	0.12
SWHAS_4604_MRG	1745	4.57	11.17	16323.51	9.052	2.461	9.98
SWHAS_472_MRG	9883	0.80	14.79	1.32	0.111	0.02	0.08
SWHAS_5464_MRG	9208	3.29	32.03	0.70	0.065	0.02	0
SWHAS_6717_MRG	3404	0	19.30	0.09	0	0.204	0
SWHDS_848_MRG	6043	2.63	109.91	34.61	0.08	0.165	0.10
STIN_351_ARG	3769	23.69	7.26	0.60	772.059	95.459	2.26
STIN_8476_ARG	4329	3.84	2.02	0.25	30.858	2.224	0
SWHAS_6974_ARG	4270	13.86	16.73	0.36	38.43	1.501	0.04
SWHAS_815_ARG	2443	2.12	19.53	0.32	4.226	0.484	0.04
SWHAS_4082_ARG&MRG	3729	0	5.71	0.05	0	0.054	0

Table S11. Assembly efficiency evaluation (CLC Genomics Workbench with different K-mer sets)

Coverage	5	10	30	50	100	200	500
23 K-mer	NA	0	1	5	5	5	3
35 K-mer	NA	NA	0	0	1	7	3
51 K-mer	NA	NA	NA	NA	NA	0	1

* ART linux64 Illumina_src-1.5.1 was used to make the plasmid sequences into illumine short reads with different coverage. NA means no contig can be assembly with the used parameters.

Table S12. Assembly efficiency evaluation (CLC Genomics Workbench with 23 k-mer)

Coverage	Retrieved reference plasmid
30	PC16
50	PC12, PC15, PC19, PC20, PC21
100	PC4, PC10, PC15, PC18, PC20
200	PC6, PC8, PC10, PC14, PC20
500	PC8, PC13, PC20

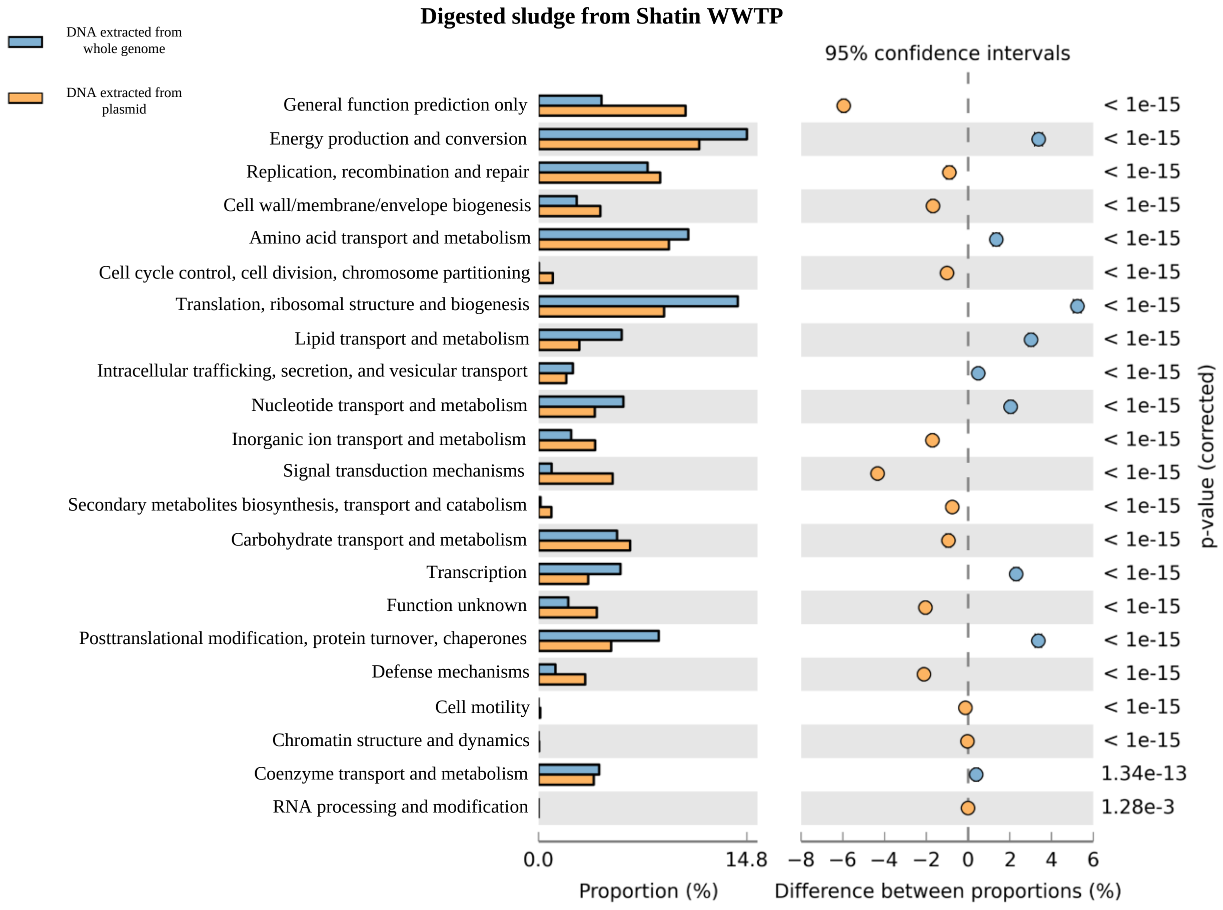

Figure S1. Functional genes in the plasmid metagenome versus the genes in the total DNA metagenome extracted from STDS based on the percentages of COG categorized genes and pairwise proportional differences calculated using STAMP.

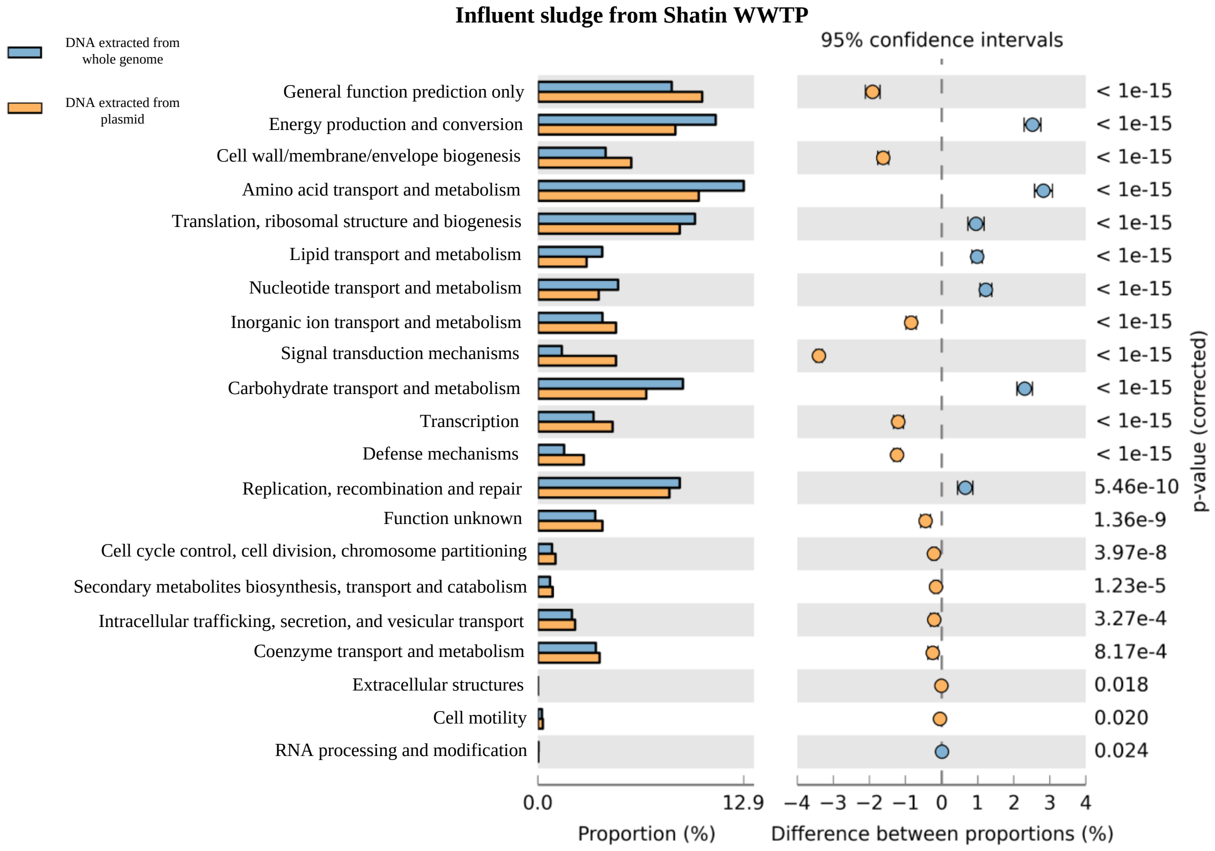

Figure S2. Functional genes in the plasmid metagenome versus the genes in the total DNA metagenome extracted from STIN based on the percentages of COG categorized genes and pairwise proportional differences calculated using STAMP..

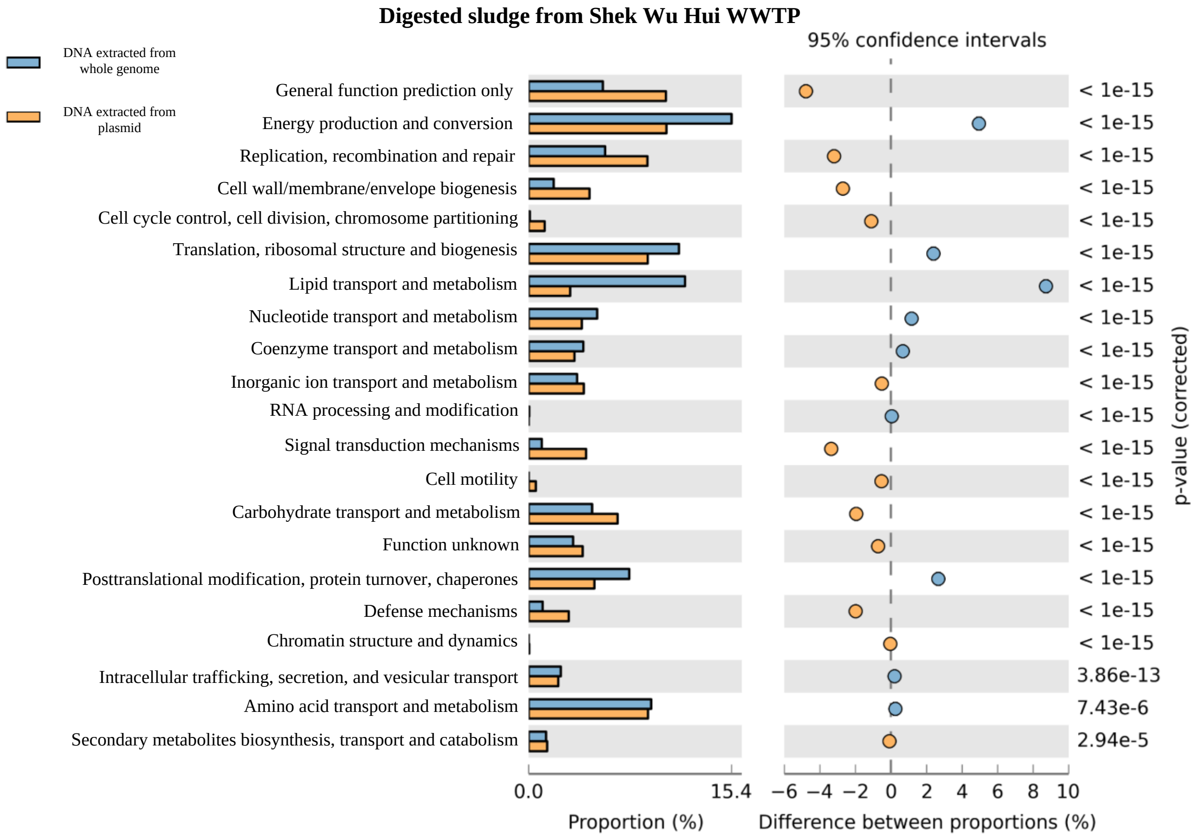

Figure S3. Functional genes in the plasmid metagenome versus the genes in the total DNA metagenome extracted from SWHDS based on the percentages of COG categorized genes and pairwise proportional differences calculated using STAMP.

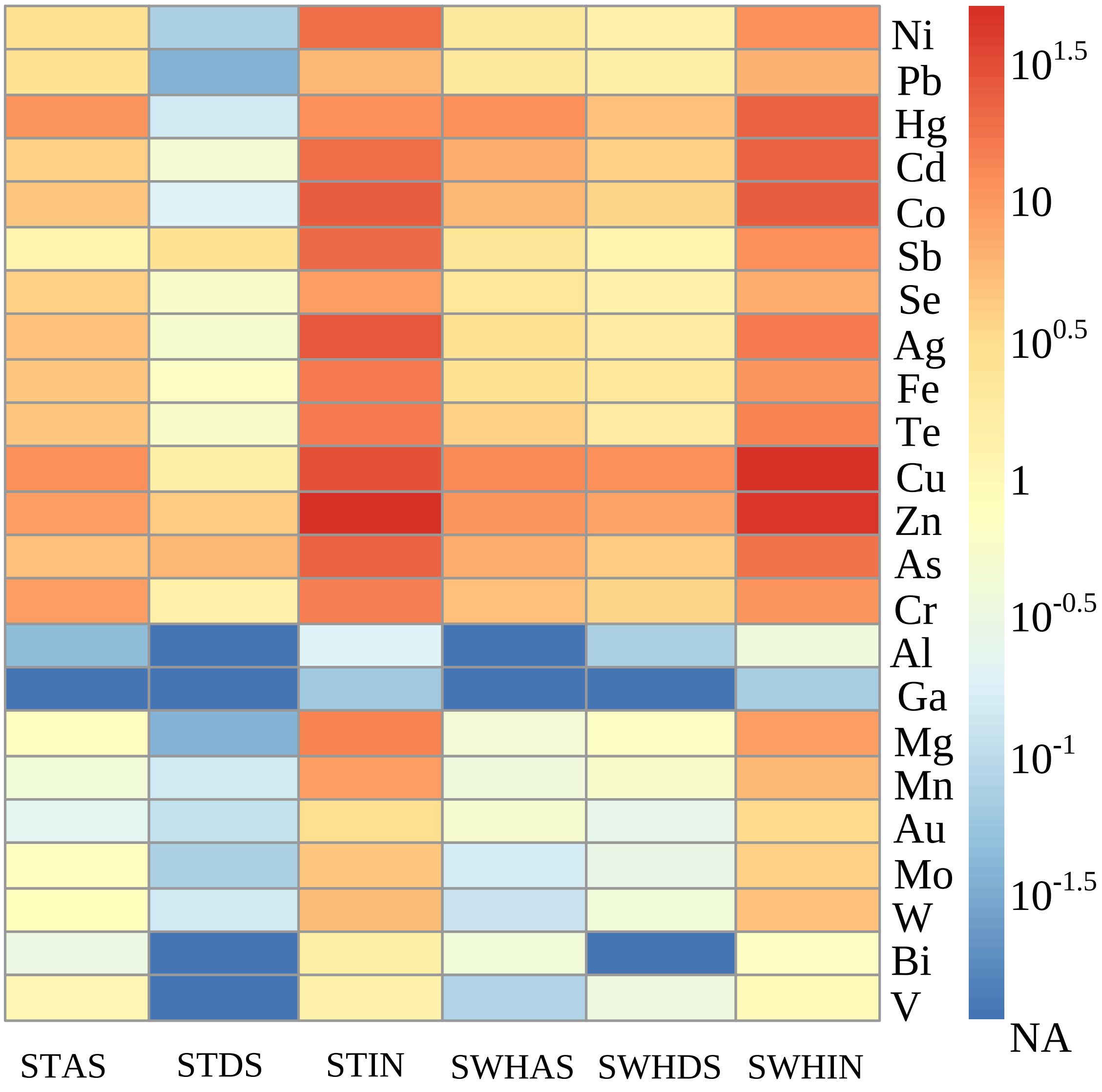

Figure S4. Distributions and abundance of each MRGs sub-type in total annotated functional sequences in the six plasmid metagenomic data sets. Every lattice represents the abundance (ppm) of the MRG-like reads.

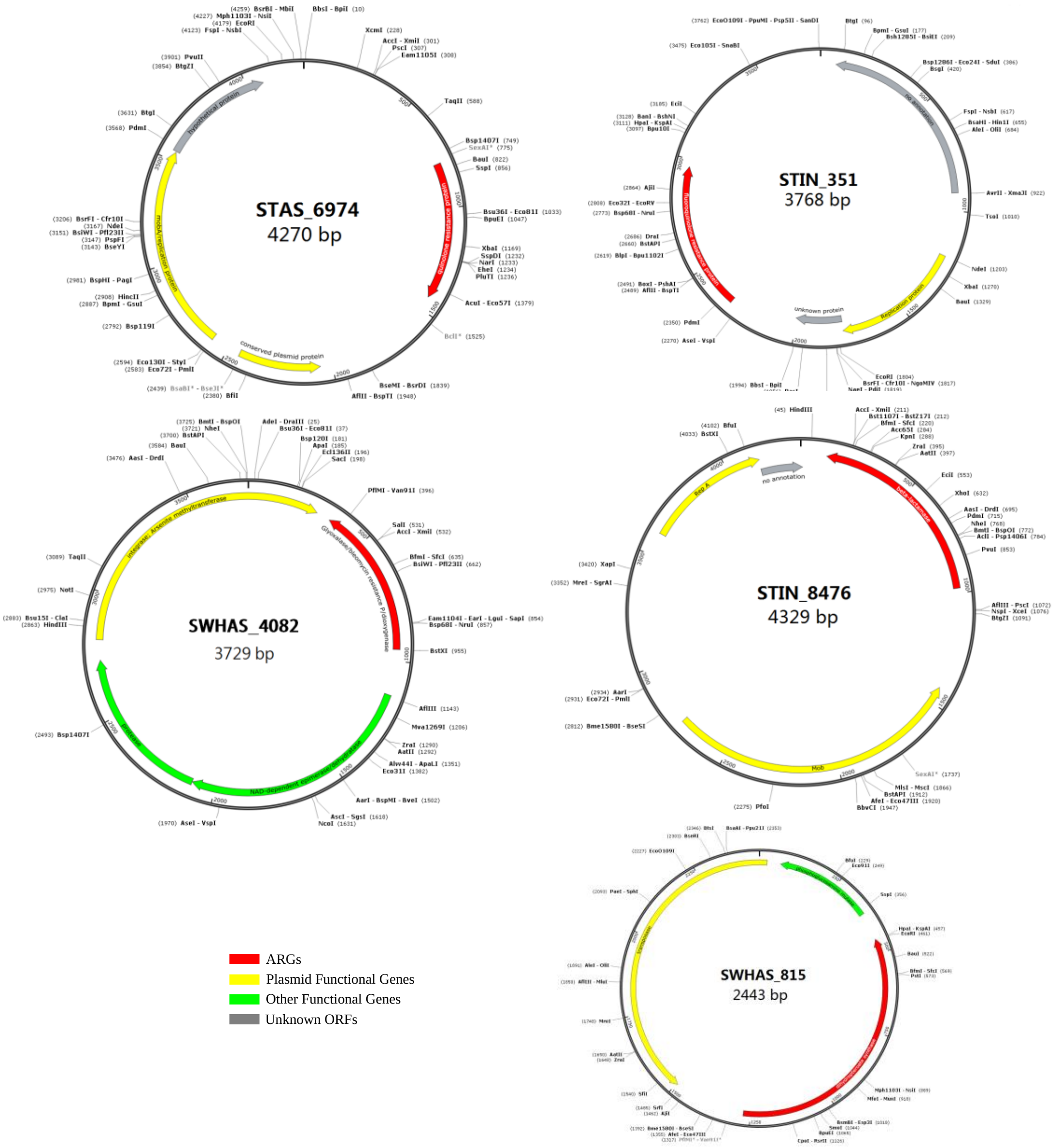

Figure S5. Maps of the potential plasmid contigs carrying ARGs.

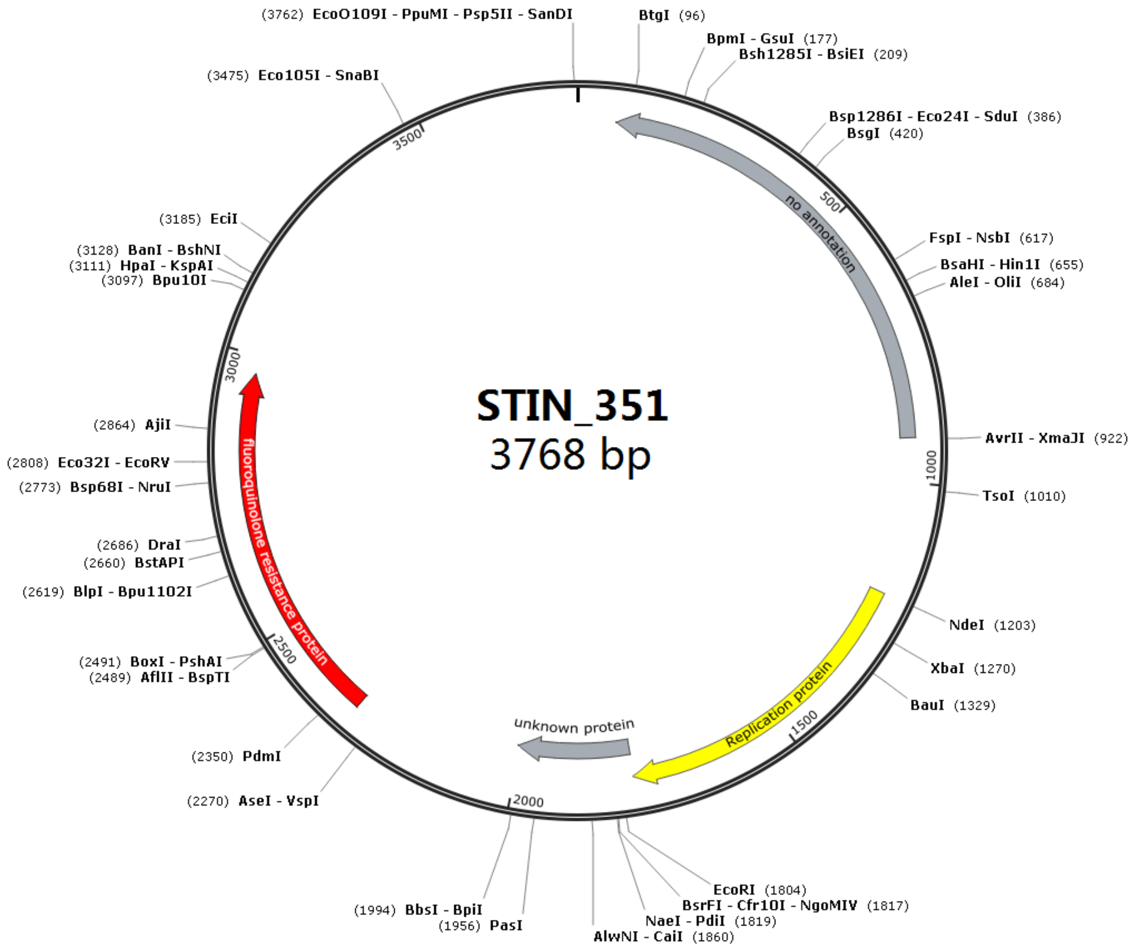

Figure S6. Map of potential plasmid STIN_351 (ARG).

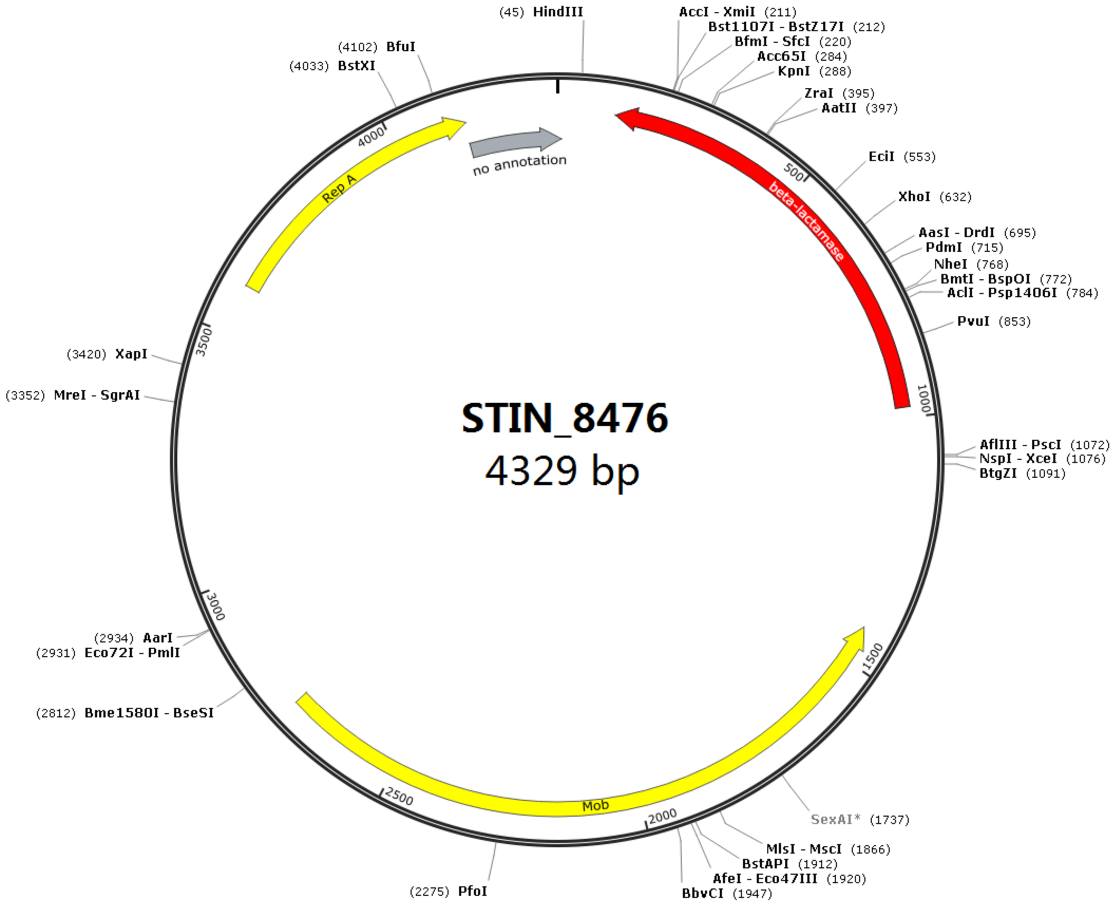

Figure S7. Map of potential plasmid STIN_8476 (ARG).

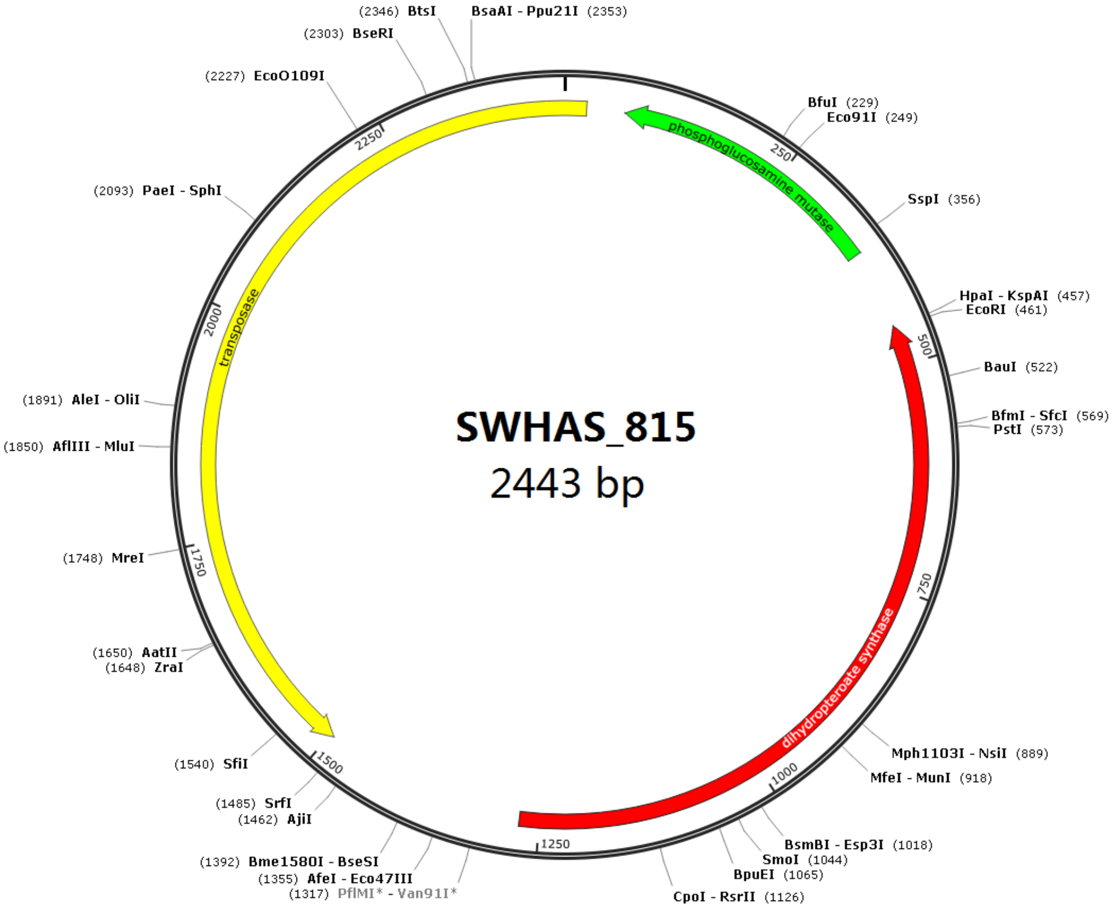

Figure S8. Map of potential plasmid SWHAS_815 (ARG).

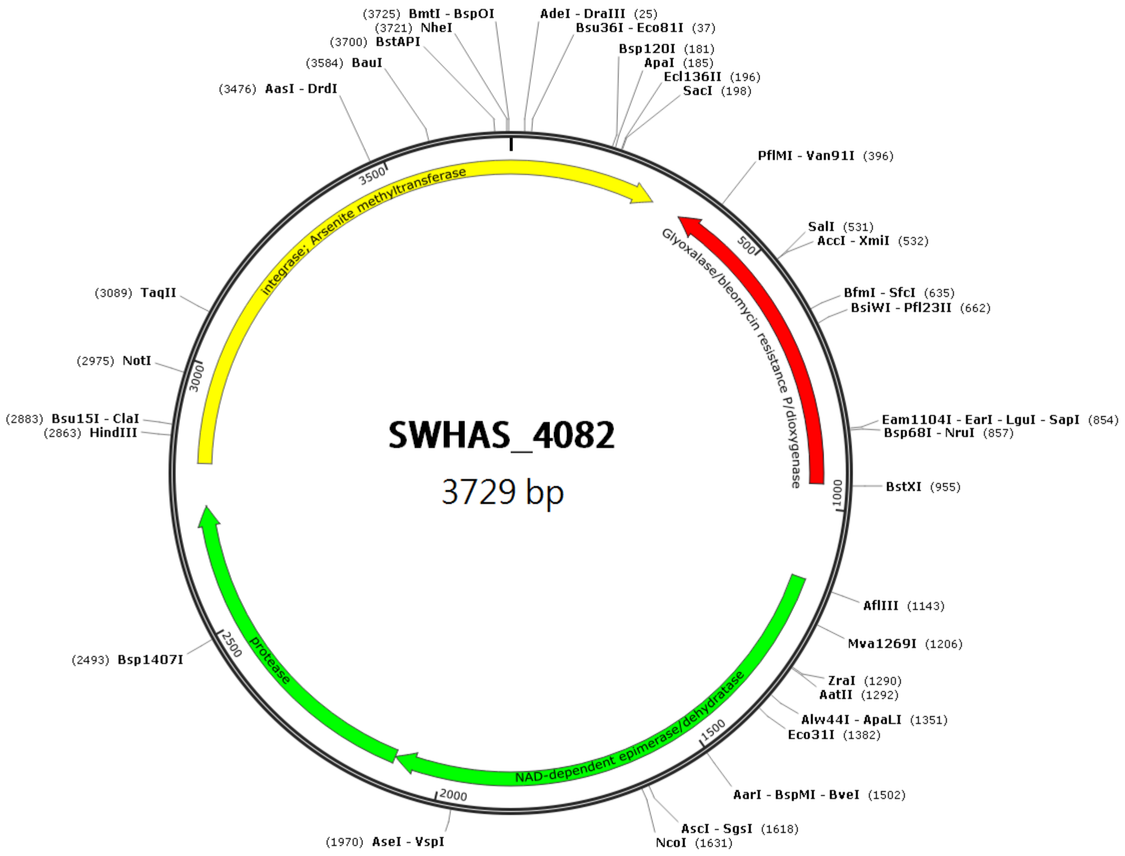

Figure S9. Map of potential plasmid SWHAS_4082 (ARG).

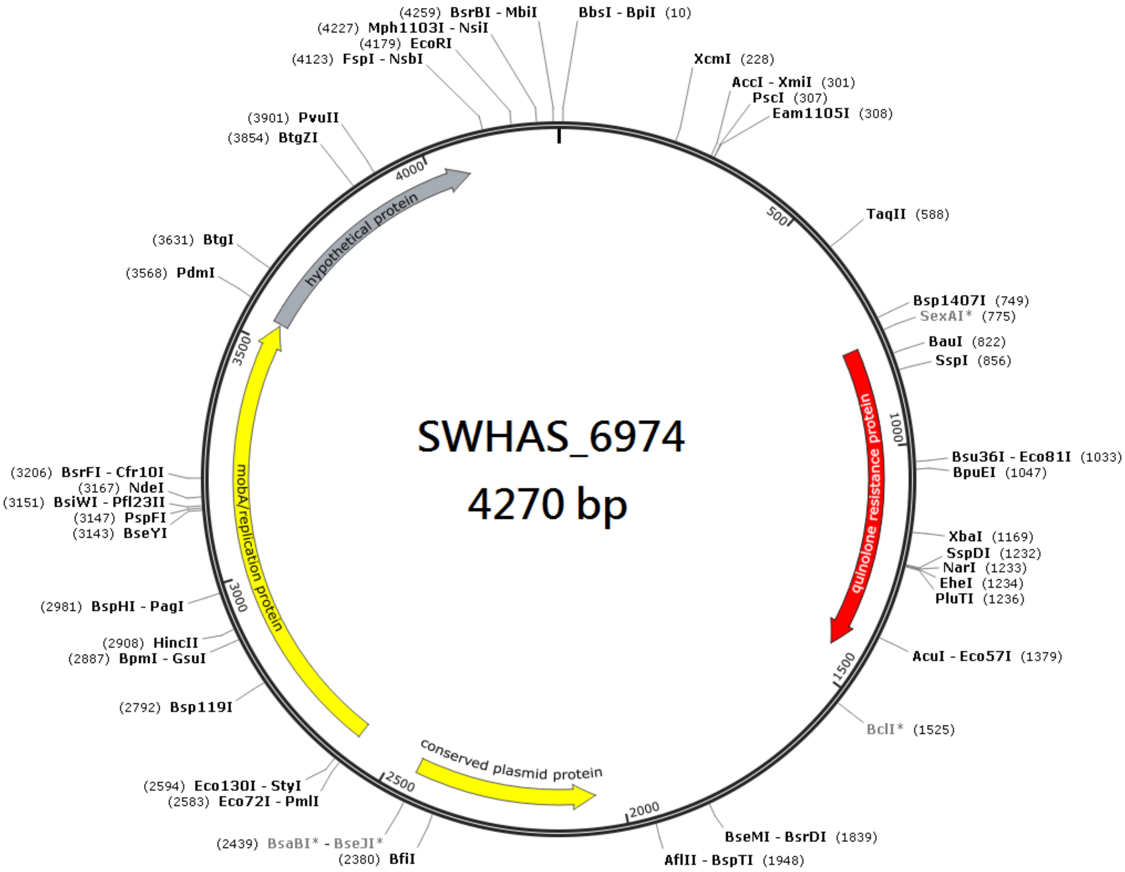

Figure S10. Map of potential plasmid SWHAS_6974 (ARG).

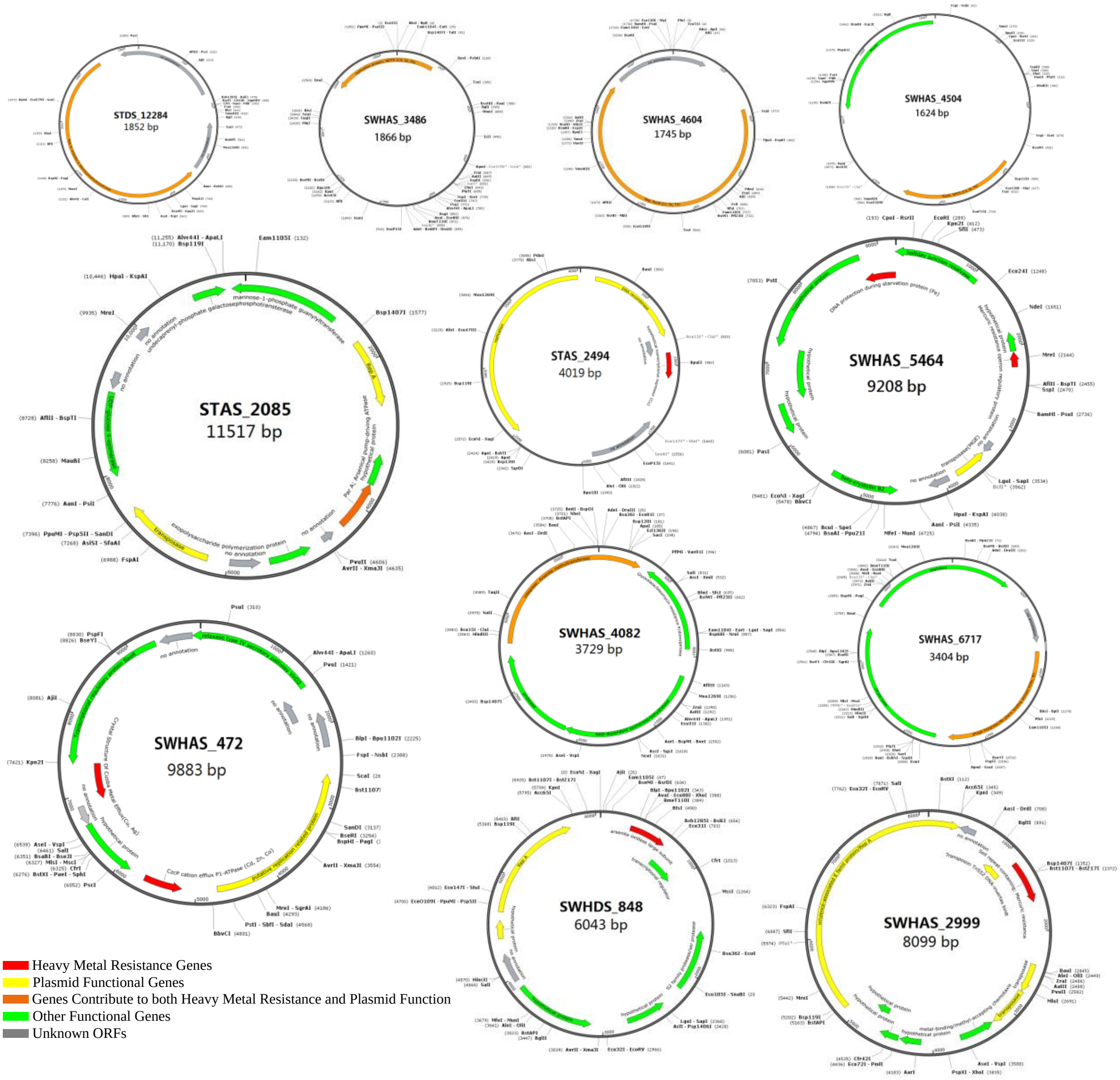

Figure S11. Maps of the potential plasmid contigs carrying MRGs.

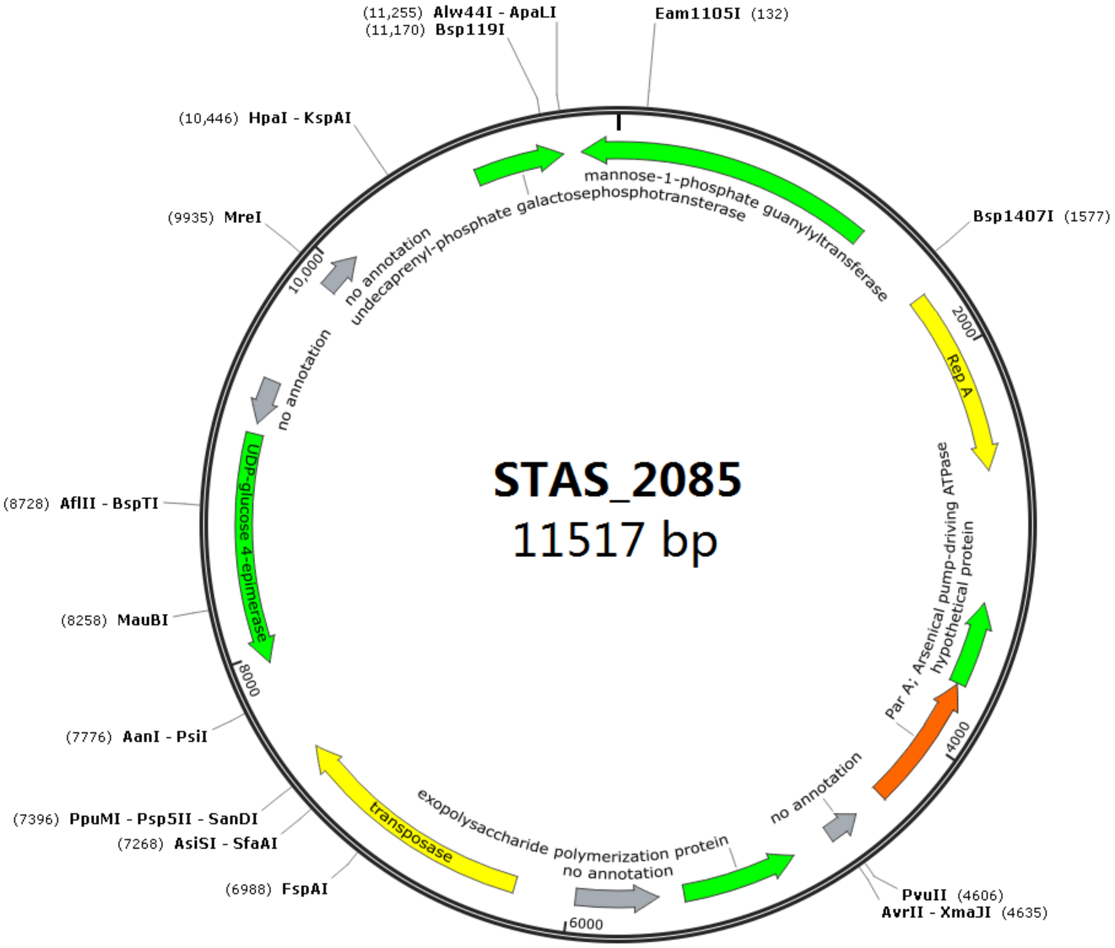

Figure S12. Map of potential plasmid STAS_2085 (MRG).

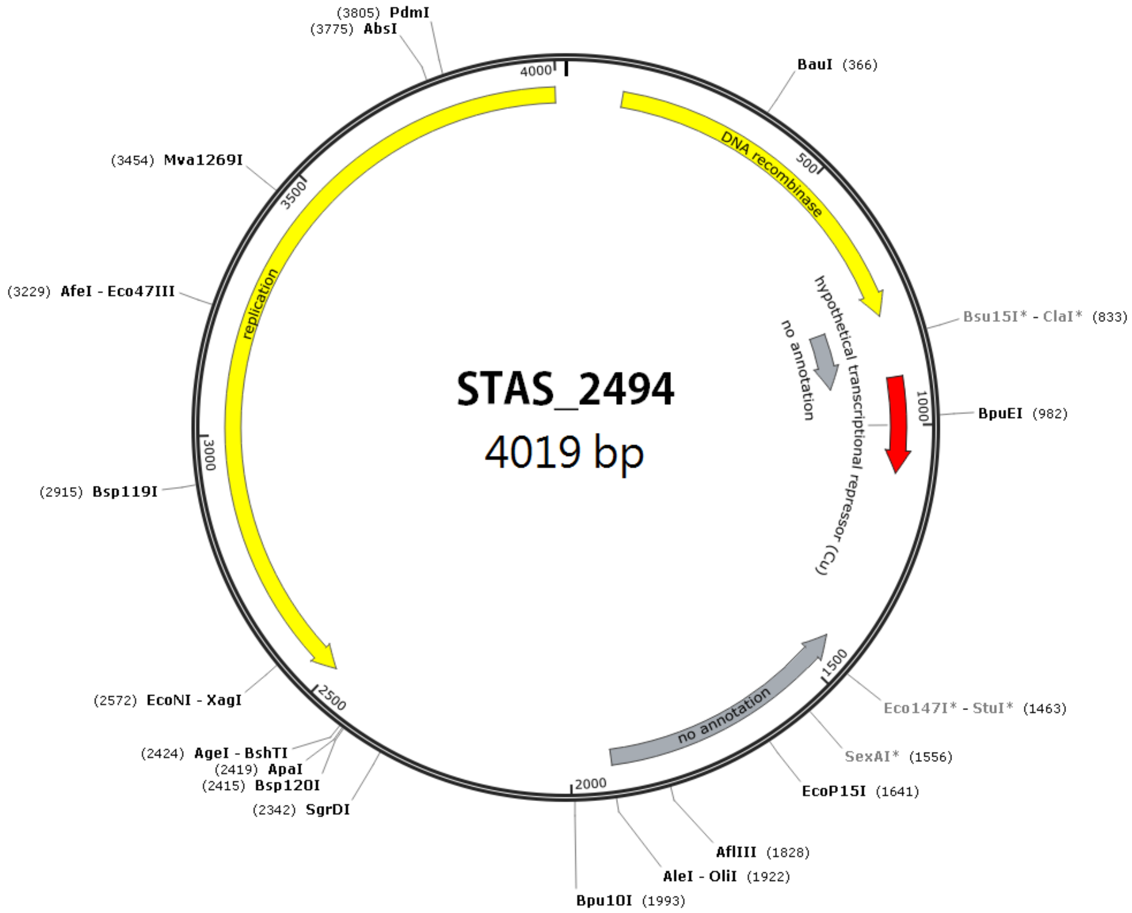

Figure S13. Map of potential plasmid STAS_2494 (MRG).

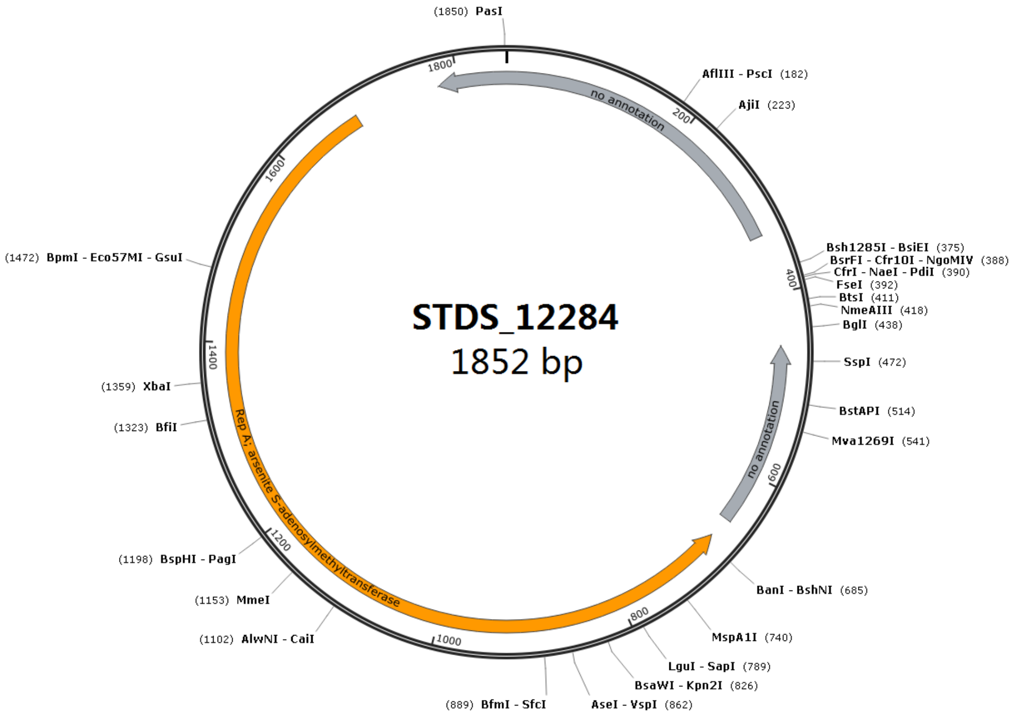

Figure S14. Map of potential plasmid STDS_12284 (MRG).

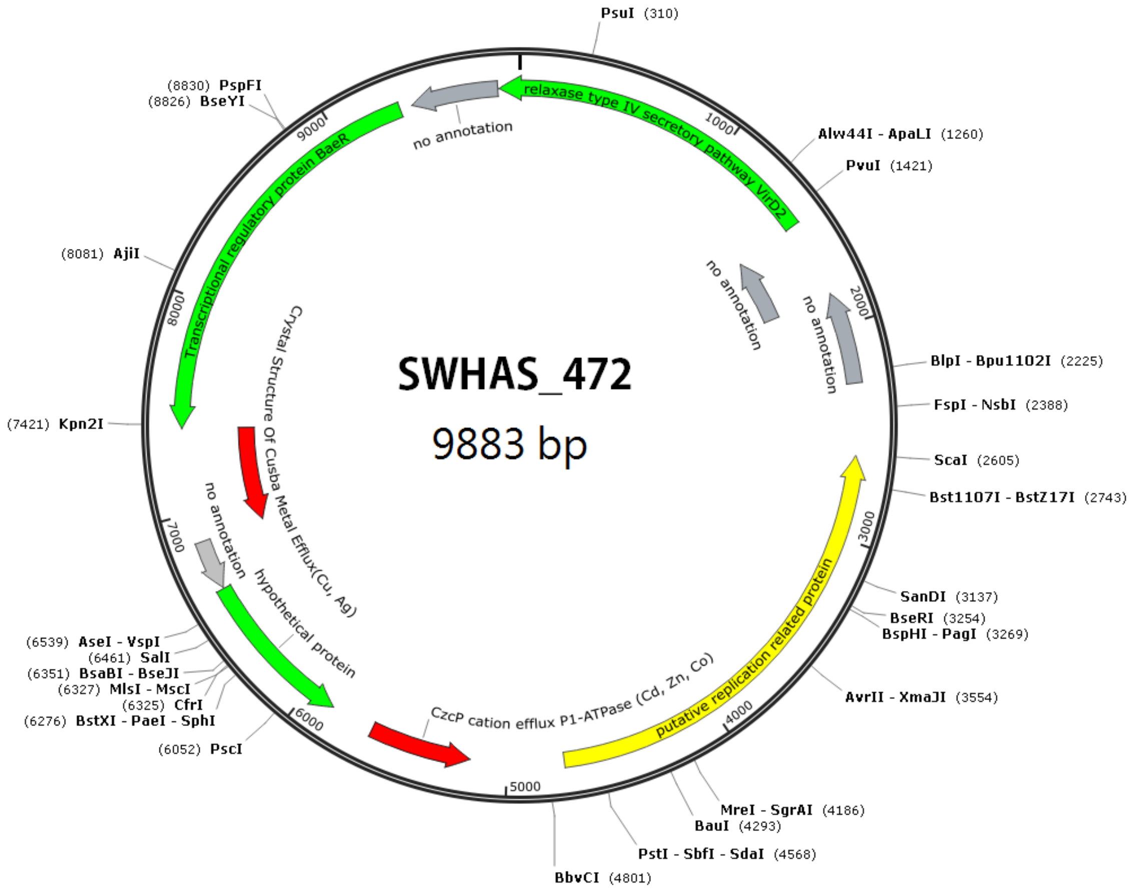

Figure S15. Map of potential plasmid SWHAS_472 (MRG).

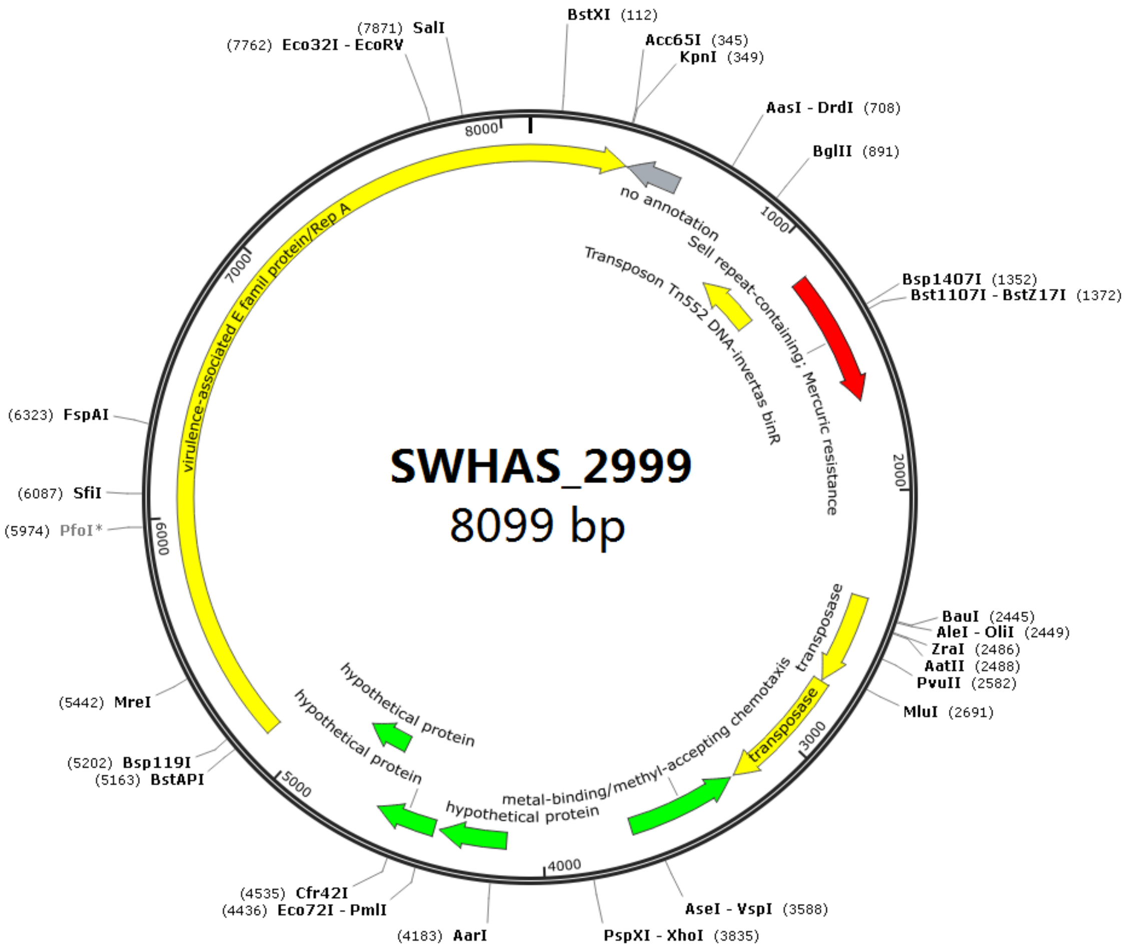

Figure S16. Map of potential plasmid SWHAS_2999 (MRG).

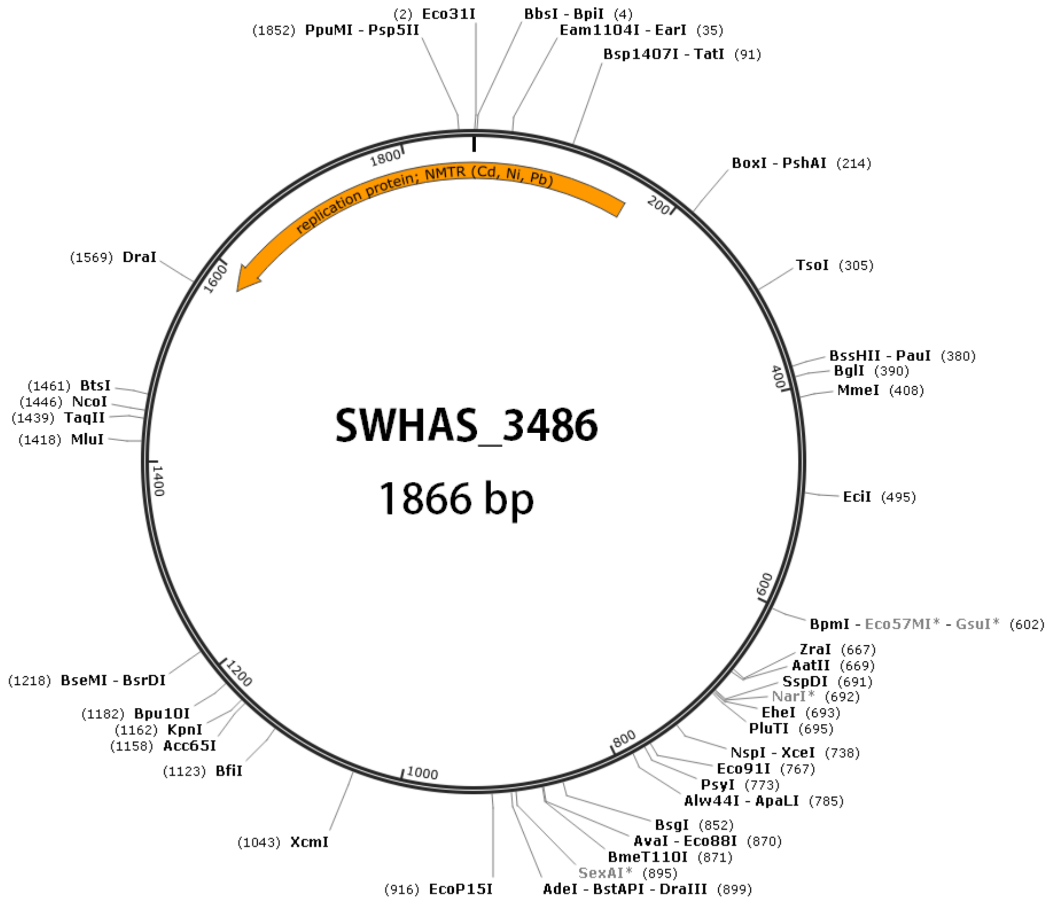

Figure S17. Map of potential plasmid SWHAS_3486 (MRG).

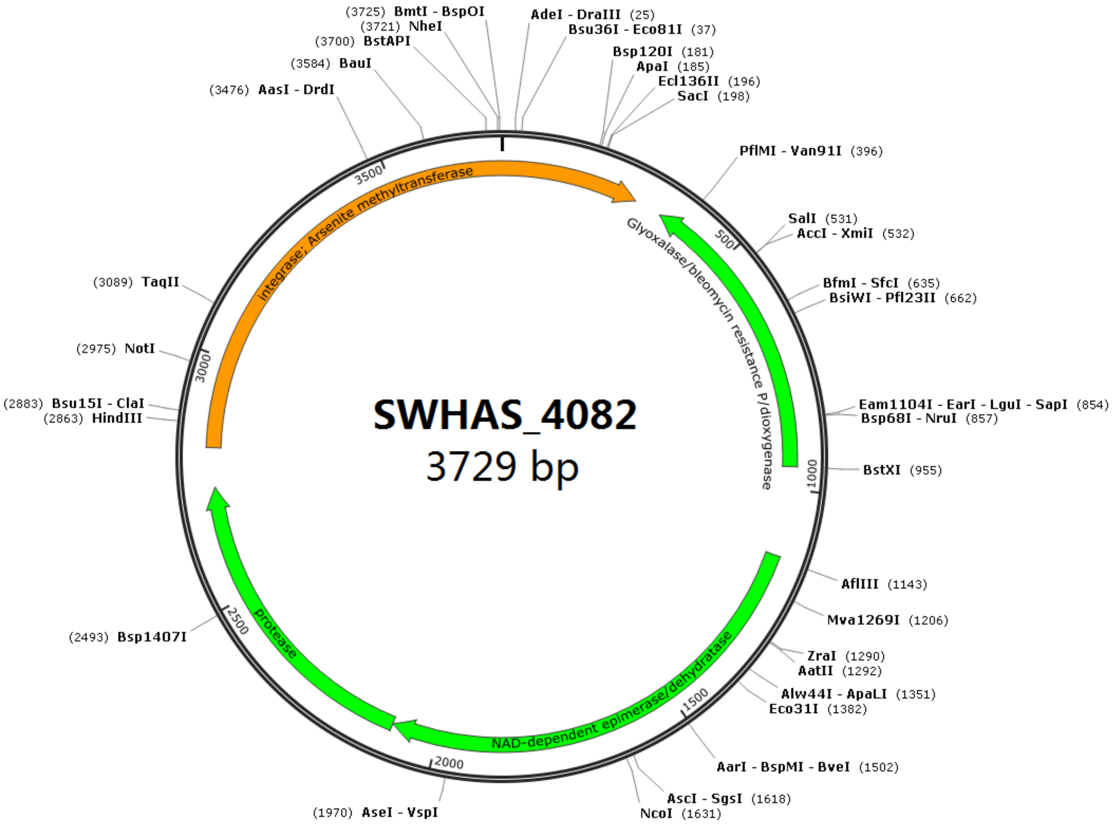

Figure S18. Map of potential plasmid SWHAS_4082 (MRG).

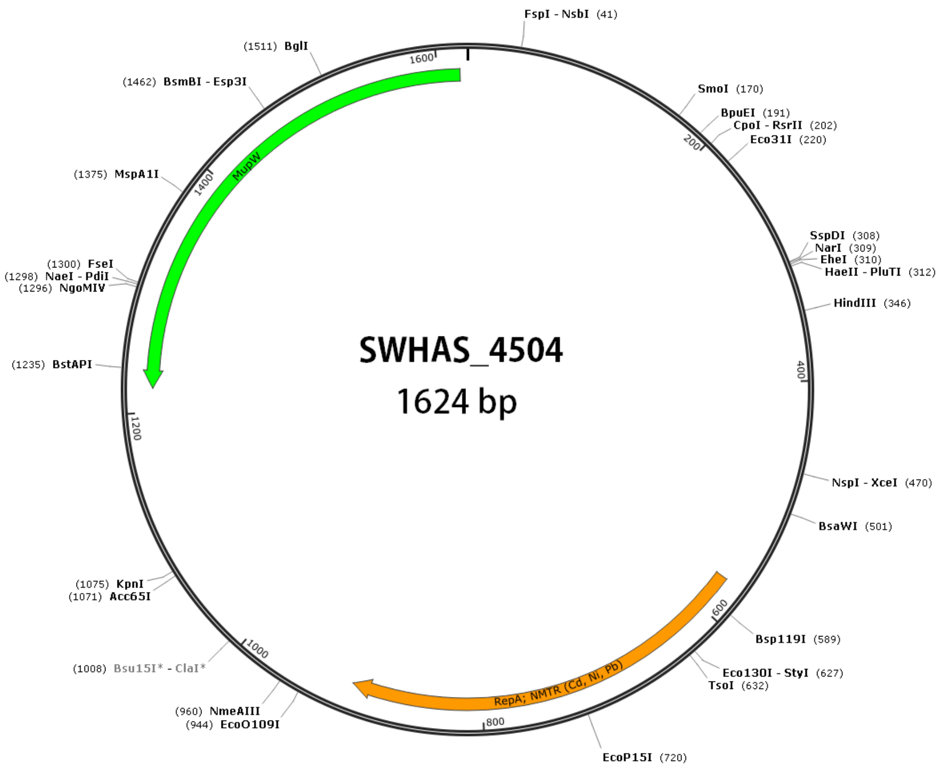

Figure S19. Map of potential plasmid SWHAS_4504 (MRG).

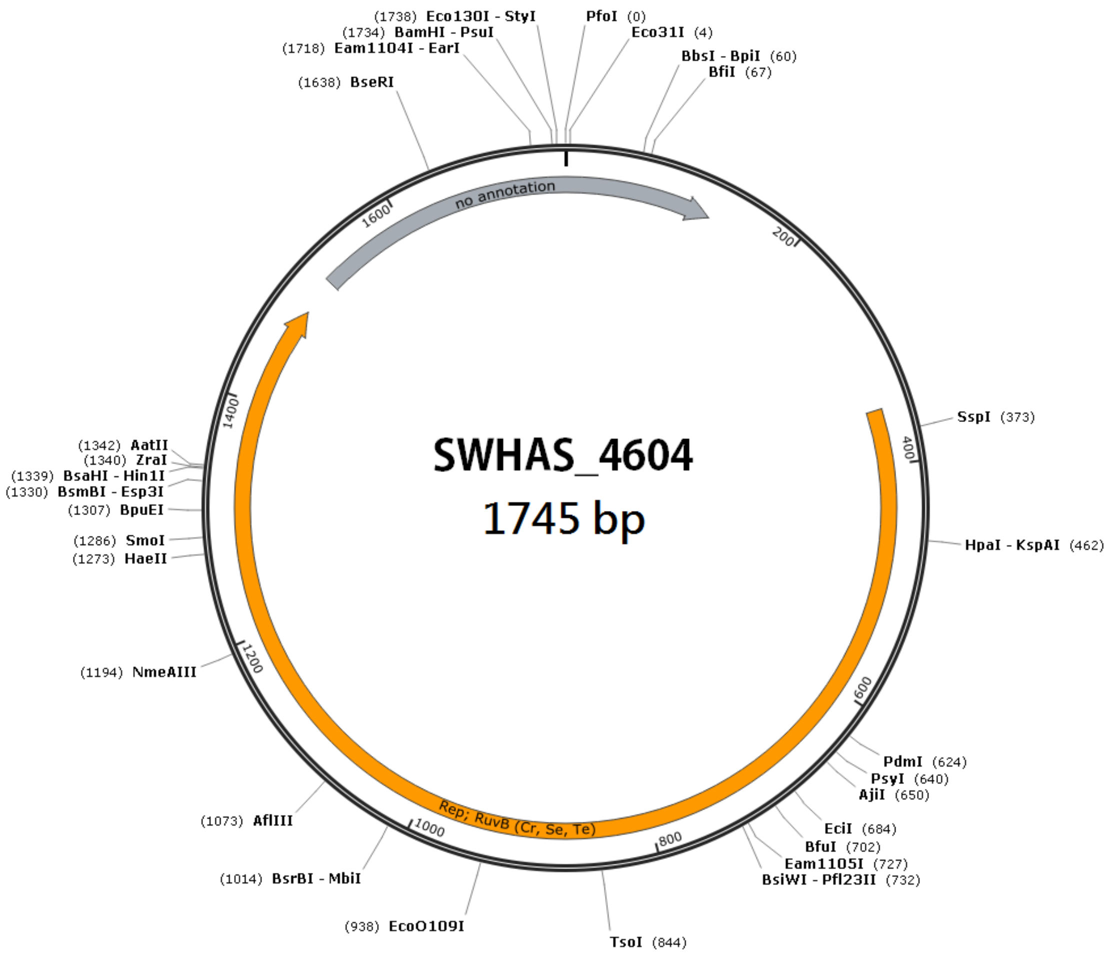

Figure S20. Map of potential plasmid SWHAS_4604 (MRG).

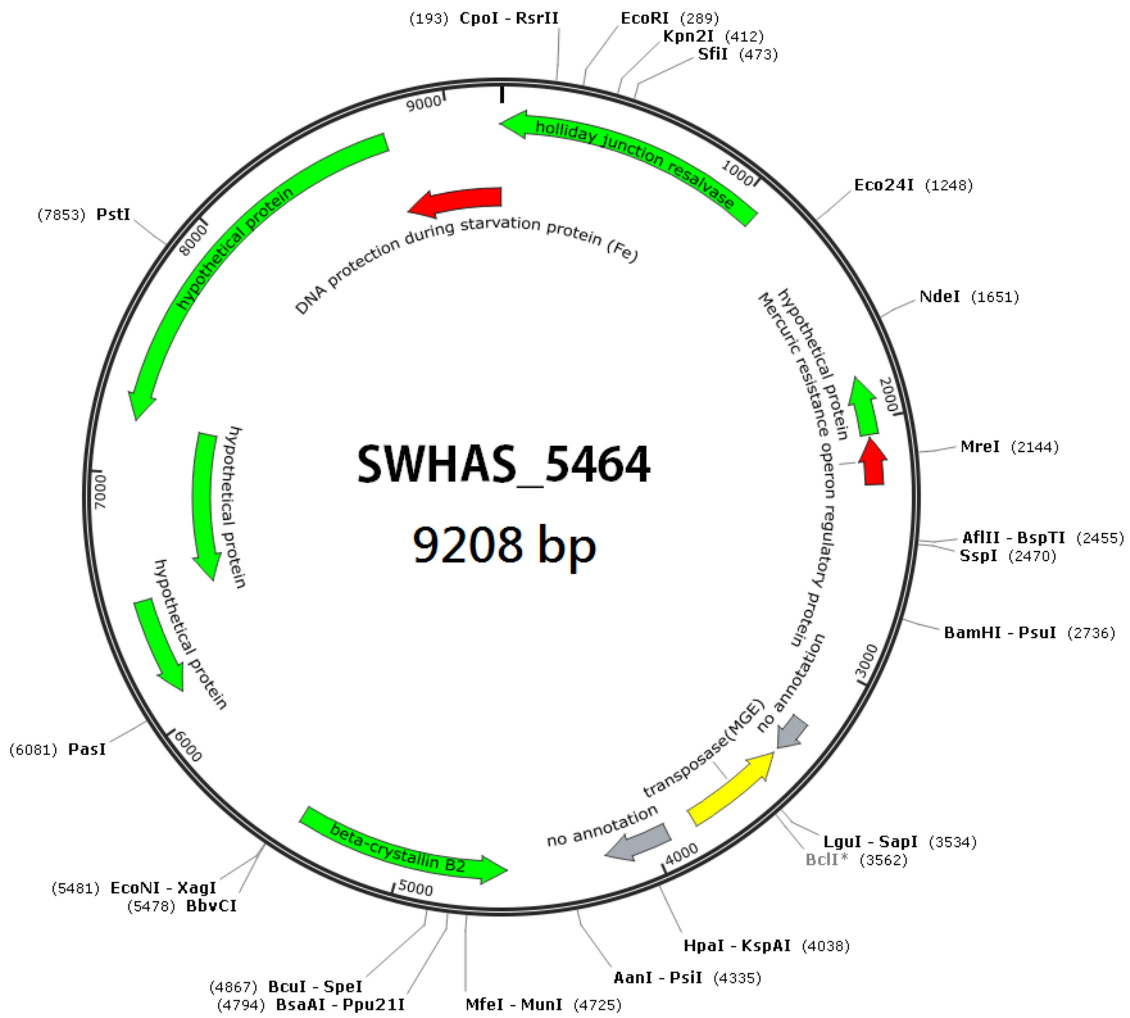

Figure S21. Map of potential plasmid SWHAS_5464 (MRG).

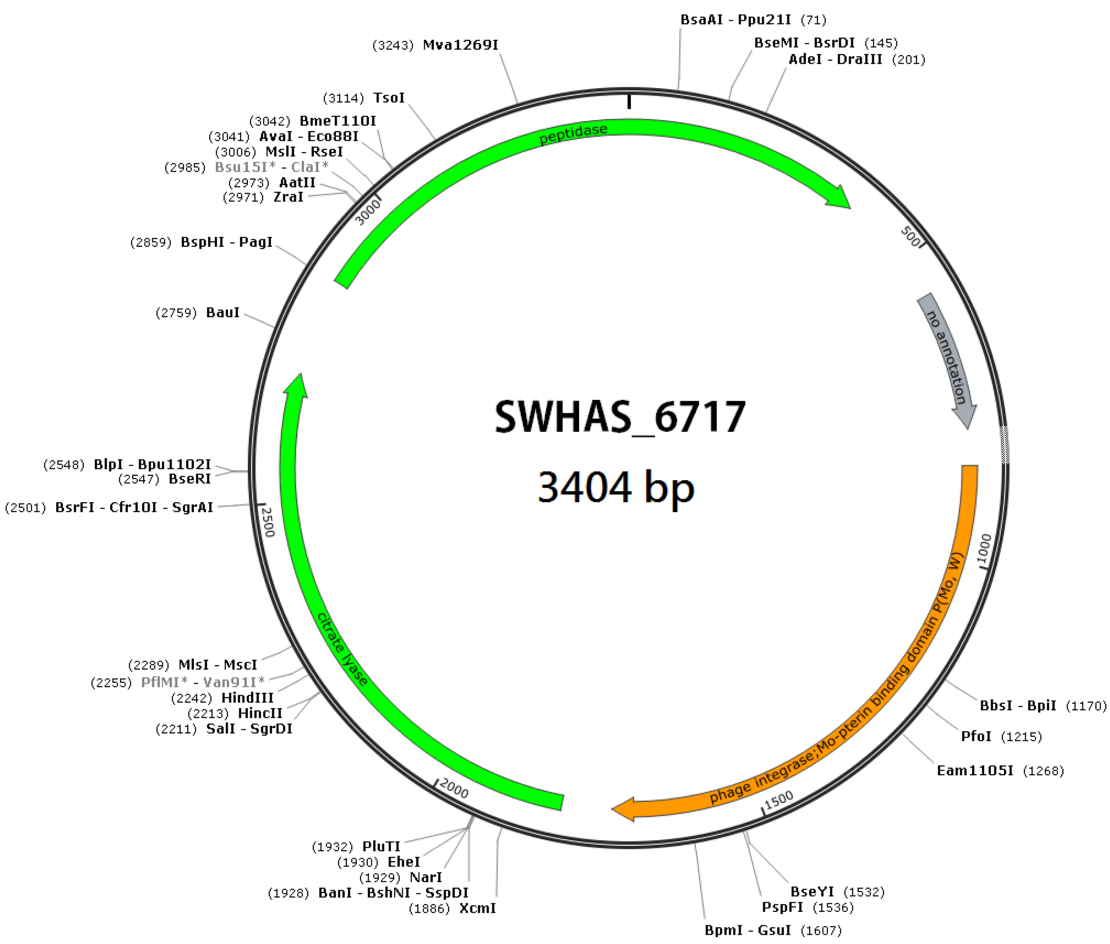

Figure S22. Map of potential plasmid SWHAS_6717 (MRG).

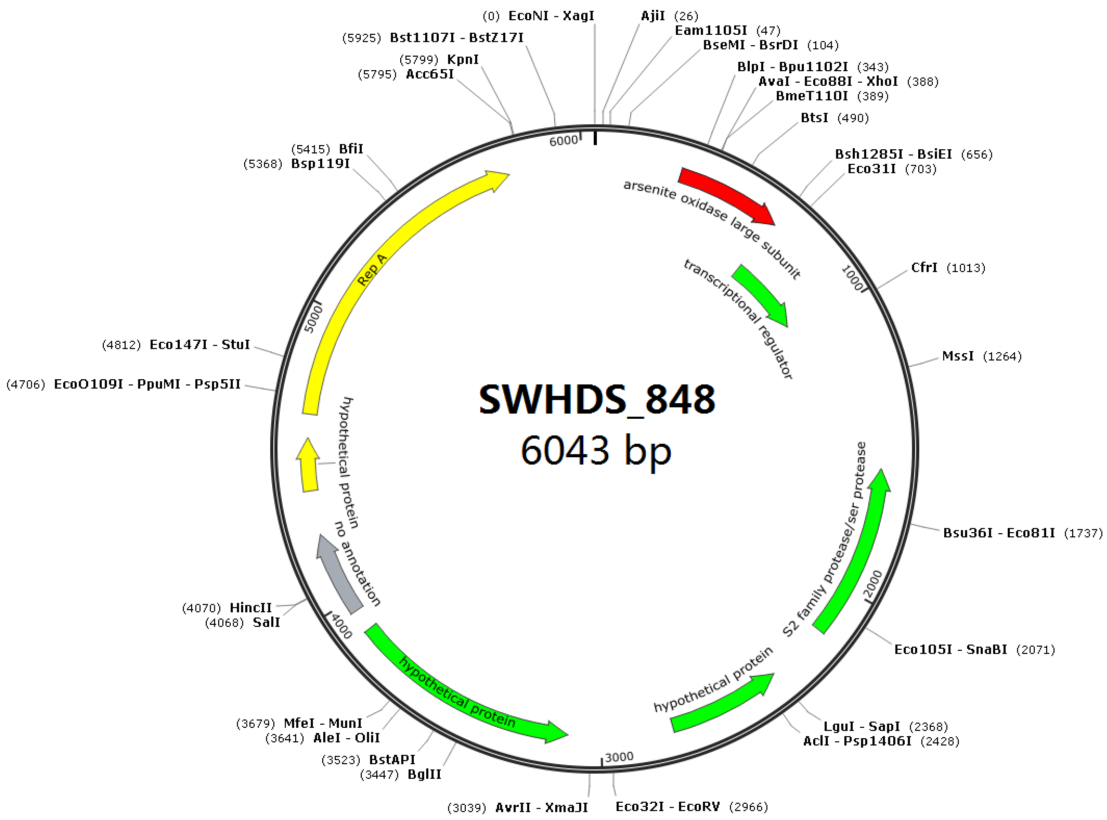

Figure S23. Map of potential plasmid SWHDS_848 (MRG).
